# The First Ring Enlargement Induced Large Piezoelectric Response in a Polycrystalline Molecular Ferroelectric

**DOI:** 10.1002/advs.202302426

**Published:** 2023-06-16

**Authors:** Yong Ai, Peng‐Fei Li, Xiao‐Gang Chen, Hui‐Peng Lv, Yan‐Ran Weng, Yu Shi, Feng Zhou, Ren‐Gen Xiong, Wei‐Qiang Liao

**Affiliations:** ^1^ Ordered Matter Science Research Center Nanchang University Nanchang 330031 P. R. China

**Keywords:** ferroelectricity, materials science, molecular ferroelectrics, multiaxial, piezoelectricity

## Abstract

Inorganic ferroelectrics have long dominated research and applications, taking advantage of high piezoelectric performance in bulk polycrystalline ceramic forms. Molecular ferroelectrics have attracted growing interest because of their environmental friendliness, easy processing, lightweight, and good biocompatibility, while realizing the considerable piezoelectricity in their bulk polycrystalline forms remains a great challenge. Herein, for the first time, through ring enlargement, a molecular ferroelectric 1‐azabicyclo[3.2.1]octonium perrhenate ([3.2.1‐abco]ReO_4_) with a large piezoelectric coefficient *d*_33_ up to 118 pC/N in the polycrystalline pellet form is designed, which is higher than that of the parent 1‐azabicyclo[2.2.1]heptanium perrhenate ([2.2.1–abch]ReO_4_, 90 pC/N) and those of most molecular ferroelectrics in polycrystalline or even single crystal forms. The ring enlargement reduces the molecular strain for easier molecular deformation, which contributes to the higher piezoelectric response in [3.2.1‐abco]ReO_4_. This work opens up a new avenue for exploring high piezoelectric polycrystalline molecular ferroelectrics with great potential in piezoelectric applications.

## Graphical abstract

A high piezoelectric molecular ferroelectric 1‐azabicyclo[3.2.1]octonium perrhenate ([3.2.1‐abco]ReO_4_) is precisely designed by the novel chemical design strategy of ring enlargement. Intriguingly, its polycrystalline pellet can show a piezoelectric coefficient *d*_33_ as large as 118 pC/N, larger than that of the parent 1‐azabicyclo[2.2.1]heptanium perrhenate ([2.2.1–abch]ReO_4_) and those of most polycrystalline or even single‐crystal molecular ferroelectrics.

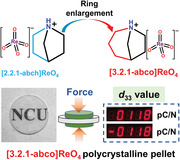

## 1 Introduction

Ferroelectrics are a kind of important functional materials with versatile physical properties such as ferroelectricity, pyroelectricity, piezoelectricity, and nonlinear optical activity.^[^ ^1^ ^]^ Among them, one of the most valuable functionalities is the piezoelectricity that enables the direct conversion between electrical and mechanical energy, which allows ferroelectrics to be widely used in high‐voltage sources, sensors, transducers, and actuators as well as the recently developed piezoelectric catalysis.^[^ ^2^ ^]^ In terms of practical applications, inorganic ferroelectrics have long dominated the mainstream due to their high piezoelectric performances and the key benefit that they can be used in bulk polycrystalline forms, e.g., ceramics, as represented by Pb(Zr,Ti)O_3_ with piezoelectric coefficient *d*_33_ up to 200–750 pC/N.^[^ ^3^ ^]^ Inorganic ferroelectric ceramics can be processed into any desired shape from solid powders by high‐temperature sintering.^[^ ^4^ ^]^ In recent years, molecular ferroelectric crystals have been gaining growing attention as potential alternatives or beneficial complements to inorganic ones, due to their advantages of environmental friendliness, low‐toxicity, lightweight, good biocompatibility, structural diversity and adjustability.^[^ ^5^ ^]^ Various types of molecular ferroelectrics have thus far been reported, including organic–inorganic hybrids,^[^ ^6^ ^]^ pure organic crystals,^[^ ^7^ ^]^ organic salts,^[^ ^8^ ^]^ organic co‐crystals,^[^ ^9^ ^]^ *etc*. some of which exhibited high piezoelectric performances that are comparable to or even better than those of inorganic ones.^[^ ^6a‐e^ ^]^ For example, the organic–inorganic [Me_3_NCH_2_Cl]CdCl_3_ crystal shows a high *d*_33_ of 220 pC/N, exceeding that of inorganic BaTiO_3_ (190 pC/N).^[^ ^6b^ ^]^ However, ferroelectric properties of almost all molecular ferroelectrics are limited to single crystals,^[^ ^6^, ^7^, ^8^, ^9^ ^]^ which necessitates addressing the significant challenge of growing single crystals with specific suitable orientations and shapes. It is important and highly desirable to achieve considerable piezoelectricity in the bulk polycrystalline forms of molecular ferroelectrics.

Inorganic ferroelectrics can realize high performance in polycrystalline ceramic forms due to their multiaxial ferroelectricity, which enables the multiple orientation tunability of the polarization.^[^ ^8b^ ^]^ Plastic crystals, as a class of molecular materials composed of molecules with globular or quasi‐spherical structures, have a plastic mesophase between the solid and liquid phases.^[^ ^10^ ^]^ In the plastic crystal phase, organic molecules can easily isotropically rotate in the 3D lattice to bring about highly symmetric cubic crystal structures.^[^ ^11^ ^]^ Together with the low symmetric polar phases with molecular orientational ordering, this unique feature tends to generate multiaxial ferroelectricity, allowing for changes in the direction of polar axis and offering the possibility for effective ferroelectric polarization switching in polycrystalline forms.^[^ ^8^, ^12^ ^]^ Equally importantly, different from other types of molecular crystals that are brittle and fragile to only form loosely bound aggregates of crystal grains, plastic crystals have good malleability and can extend without cracking, enabling the easy preparation of monolithic bulk polycrystals by pressing powders^[^ ^13^ ^]^ and favoring the autonomous self‐healing in some molecular piezoelectric crystals.^[^ ^14^ ^]^ Remarkable progress in plastic ferroelectric crystals has been made in the past few years,^[^ ^11^, ^12^, ^13^, ^15^ ^]^ some of which bulk polycrystalline forms can show substantial piezoelectricity.^[^ ^15^ ^]^ For instance, [C(NH_2_)_3_]ClO_4_ and [(CH_3_)_4_N][GaCl_4_] polycrystalline ferroelectric was found to show a *d*_33_ of 10 and 80 pC/N, respectively.^[^ ^15a,b^ ^]^ Harada et al. reported the piezoelectric response of several polycrystalline molecular ferroelectrics including guanidinium tetrafluoroborate (*d*_33_ = 18 pC/N),^[^ ^15c^ ^]^ [(CH_3_)_4_N][FeCl_4_] (*d*_33_ = 80 pC/N),^[^ ^15d^ ^]^ 1‐azabicyclo[2.2.1]heptanium perrhenate ([2.2.1‐abch]ReO_4_, *d*_33_ = 90 pC/N).^[^ ^15e^ ^]^ However, high‐piezoelectric‐performance polycrystalline molecular ferroelectrics are still very rare. Moreover, although the strategies for obtaining high piezoelectricity in molecular ferroelectric single crystals have been well developed, such as the bond‐weakening approach and heavy halogen substitution,^[^ ^1^, ^16^ ^]^ it remains a great challenge to design polycrystalline molecular ferroelectrics with high piezoelectricity.

**Scheme 1:**
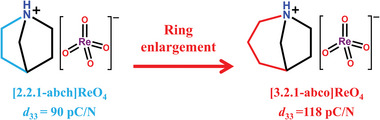
Molecular modification strategy of ring enlargement for designing high piezoelectric polycrystalline molecular ferroelectric [3.2.1‐abco]ReO_4_.

Here, through the chemical design strategy of enlarging the ring of 1‐azabicyclo[2.2.1]heptanium cation in [2.2.1‐abch]ReO_4_ (**Scheme** **1**), we designed a new molecular ferroelectric 1‐azabicyclo[3.2.1]octanium perrhenate ([3.2.1‐abco]ReO_4_), which is able to show high piezoelectric performance in polycrystalline pellet form, behaving like to the inorganic ferroelectric ceramics. Benefit from the ring enlargement reduced molecular strain, the [3.2.1‐abco]ReO_4_ polycrystalline pellet exhibits a remarkably large *d*_33_ up to 118 pC/N, which is higher than that of the parent [2.2.1‐abch]ReO_4_ (90 pC/N) (Scheme 1) and those of most molecular ferroelectrics in polycrystalline or even single‐crystal forms. To our knowledge, [3.2.1‐abco]ReO_4_ is the first example of ring enlargement obtained polycrystalline molecular ferroelectric with large piezoelectric response. This work provides an efficient route to design high piezoelectric polycrystalline molecular ferroelectrics.

## 2 Results and Discussion

**Figure 1:**
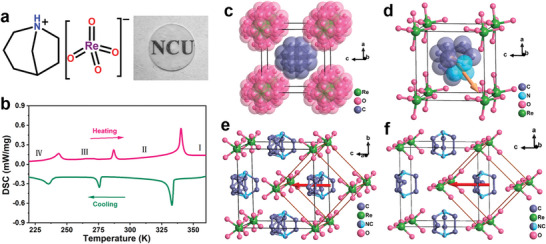
a) Molecular formula of [3.2.1‐abco]ReO_4_ and its translucent polycrystalline pellet. b) DSC curves of [3.2.1‐abco]ReO_4_. c–f) Crystal structures of [3.2.1‐abco]ReO_4_ in c) phase I, d) phase II, e) phase III, and f) phase IV. The brown lines in Figure 1e–f represent the unit cell of phase I. The gold and red arrows represent the polar axis direction.

[3.2.1‐Abco]ReO_4_ was easily prepared as colorless block single crystals by a slow evaporating methanol solution consisting of equimolar 1‐azabicyclo[3.2.1]octane and perrhenic acid. The phase purity of as‐grown crystals of [3.2.1‐abco]ReO_4_ was confirmed by the powder X‐ray diffraction measurements (Figure S1, Supporting Information). Its molecular formula and prepared translucent polycrystalline pellet are shown in **Figure** **1**a. Differential scanning calorimetry (DSC) measurements were performed on powdered crystalline samples, which reveal that [3.2.1‐abco]ReO_4_ undergoes three solid−solid reversible phase transitions at 340, 286 and 243 K, respectively (Figure 1b). For the convenience of description, we label the four solid phases separated by the three transitions as I–IV phases, with the phase above 340 K represented as phase I and the subsequent lower temperature phases as phases II‐IV. Thermogravimetric analysis (TGA) shows that [3.2.1‐abco]ReO_4_ has good thermal stability with a decomposition temperature up to 630 K (Figure S2, Supporting Information).

Single‐crystal X‐ray diffraction analyses were performed to investigate the phase transition mechanism and ferroelectric origin, and the related crystal data for [3.2.1‐abco]ReO_4_ are summarized in Table S1 (Supporting Information). At 373 K in phase I, [3.2.1‐abco]ReO_4_ crystallizes in the *Pm* $\bar{3}$ *m* space group with cubic unit cell parameters (*a* = 6.4362(3) Å, *V* = 266.62(4) Å^3^), belonging to the cubic crystal system and the *m* $\bar{3}$ *m* Laue class. Both cations and anions are severely disordered in this highest symmetric site, showing structurally isotropic tumbling and losing chemical sense (Figure 1c). The cubic crystal symmetry and the fully disordered molecular orientations strongly indicate the plastic characteristics in phase I. With the temperature entering into phase II, the crystal structure of [3.2.1‐abco]ReO_4_ belongs to the trigonal crystal system with a pseudocubic rhombohedral unit cell (*a* = 6.3552(8) Å and *α* = 89.73°, V = 256.67(10) Å^3^ at 300 K, space group *R*3*m*), where the cations and anions are still orientationally disordered (Figure 1d). It is noteworthy that all the [3.2.1‐abco]^+^ cations are aligned in a head‐to‐tail manner along the longer body diagonal direction of the crystal cell (Figure S3, Supporting Information), which coincides exactly with the crystallographic 3‐fold axis. Despite the cations locate on the crystallographic 3‐fold axis showing a triple orientational disorder, this aligned arrangement leads to spontaneous polarization along the 3‐fold axis, *i.e*., the [111] direction (Figure 1d), consistent with the symmetric requirement of polar 3*m* point group. All four body diagonals of the cubic lattice in phase I are equivalent 3‐fold axes, each of which corresponds to the polarization axis of the trigonal lattice in phase II. Thus, [3.2.1‐abco]ReO_4_ is a multiaxial ferroelectric crystal, whose phase II has eight equivalent polarization directions. From paraelectric phase I with *m* $\bar{3}$ *m* point group to ferroelectric phase II with 3*m* point group, the number of symmetry elements decreases from 48 to 6, which also indicates eight equivalent polarization directions in phase II.

In phase III, the crystal symmetry of [3.2.1‐abco]ReO_4_ decreases again to orthorhombic *Amm*2 space group with unit cell parameters of *a* = 6.2218(6) Å, *b* = 9.0504(6) Å, and *c* = 8.9667(7) Å at 253 K. Structurally, the cations are located on two mirror planes perpendicular to the *a*‐ and *b*‐axis directions respectively and thereby adopt a different orientation state compared to that of phase I (Figure 1e). [3.2.1‐abco]ReO_4_ in phase IV still crystallizes in the *mm*2 point group but with a *Pmn*2_1_ space group. In phase IV, the anions become completely ordered while the cations remain in an orientation disordered state (Figure 1f). In brief, the mechanism of these phase transitions is attributed to the gradually re‐orientational ordering of organic [3.2.1‐abco]^+^ cations and ReO_4_^−^ anions. With the occurrence of symmetry breaking, the polarization direction of *mm*2 phase is confined to the [110]‐direction of the cubic phase I, that is [001] direction in phases III and IV (Figure 1e,f; Figure S4, Supporting Information), thus the [3.2.1‐abco]ReO_4_ has 12 equivalent polarization directions.

**Figure 2:**
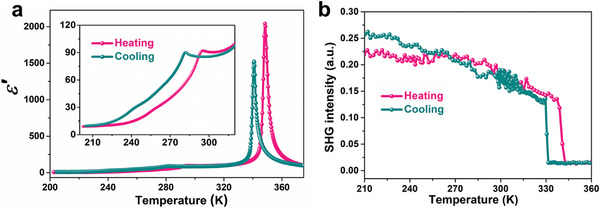
Phase transition behaviors of [3.2.1‐abco]ReO_4_. a) The real part (*ε*′) of dielectric constants as a function of temperature. b) Temperature‐dependent SHG intensity.

Solid–solid phase transition behaviors generally give rise to abnormal changes in electrical properties, i.e., anomalies in dielectric constants. **Figure** **2**a depicts the real part (*ε′*) of the complex dielectric constant of polycrystalline pellet for [3.2.1‐abco]ReO_4_. Accompanying the phase transition between phase I and II, the *ε′* shows a sharp *λ*‐shaped anomaly with a peak value up to 2041, which is a typical characteristic of paraelectric‐ferroelectric phase transitions, indicating a drastic change in the electric polarization. The temperature‐dependent *ε′* values well obeyed the Curie−Weiss law, *ε′* = *C*/(*T* − *θ*), above the *T*_C_ of 340 K (Figure S5, Supporting Information). We can obtain a linear fitting of the 1/*ε′* − *T* relationship, the deduced Curie constant C of 2977 K, and the Curie−Weiss temperature *θ* of 341 K. By comparison, the change of *ε′* value in the process of phase transition II‐III is relatively small but much larger than the common phase transitions of molecular crystals. The variation of *ε′* value during phase transition III‐IV appears to be very insignificant, which may be related to the fact that the point group has not changed. We further carried out the second harmonic generation (SHG) measurements to investigate the crystal symmetry of each phase (Figure 2b; Figure S6, Supporting Information). The SHG responses of phases I‐III are active with clear strength, consistent with the non‐centrosymmetric crystal structure (*mm*2 and 3*m*). When the temperature reaches near the Curie temperature of 340 K, the SHG intensity sharply drops into an inactive state, meaning that it enters the centrosymmetric paraelectric phase.

**Figure 3:**
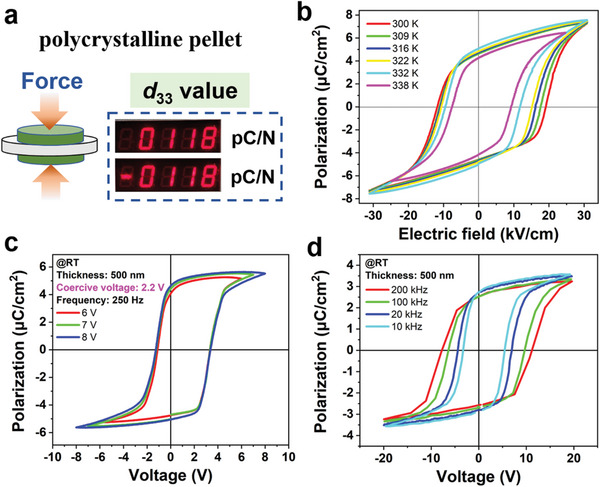
Piezoelectric and ferroelectric properties of [3.2.1‐abco]ReO_4_. a) Longitudinal piezoelectric coefficient *d*_33_ measured on the polycrystalline pellet at room temperature. b) Polarization−electric field hysteresis loops measured on polycrystalline pellet in the temperature range of 300−338 K at frequency of 50 Hz. c) Polarization−voltage hysteresis loops measured on thin film measured at room temperature under the electric field frequency of 250 Hz by applying different voltages. The thickness of measured thin film is 500 nm. d) Polarization−voltage hysteresis loops measured on thin film measured at room temperature with electric field frequencies of 10−200 kHz.

Crystals with polar structures naturally exhibit piezoelectric responses due to the lack of inversion centers. The longitudinal piezoelectric coefficient *d*_33_ is one of the most important parameters for evaluating the piezoelectric performance of materials, expressed as the induced charge per unit force generated in the direction of the applied force. Considering the potential of practical applications, we mainly focus on the piezoelectricity of its room temperature 3*m* phase, of which the piezoelectric coefficient matrix was given in Equation S1 (Supporting Information). As shown in **Figure** **3**a, excitingly, the polycrystalline pellet of [3.2.1‐abco]ReO_4_ shows remarkably large piezoelectric response with a high *d*_33_ value of 118 pC/N at room temperature after poling processing, which is higher than that of the parent [2.2.1‐abch]ReO_4_ (90 pC/N).^[^ ^15e^ ^]^ Meanwhile, such a high *d*_33_ value of 118 pC/N is among the highest one in polycrystalline molecular ferroelectric, higher than those of guanidinium tetrafluoroborate (18 pC/N),^[^ ^15c^ ^]^ [(CH_3_)_4_N][GaCl_4_] (80 pC/N),^[^ ^15b^ ^]^ [(CH_3_)_4_N][FeCl_4_] (80 pC/N),^[^ ^15d^ ^]^ and [(CH_3_)_4_N][FeBrCl_3_] (110 pC/N),^[^ ^15d^ ^]^ The *d*_33_ of [3.2.1‐abco]ReO_4_ is even larger than most single‐crystal molecular ferroelectrics including imidazolium perchlorate (41 pC/N),^[^ ^17^ ^]^ NDABCO‐NH_4_‐Br_3_ (63 pC/N),^[^ ^18^ ^]^ (RM_3_HQ)_2_NH_4_La(NO_3_)_6_ (81 pC/N),^[^ ^6c^ ^]^ (RM_3_HQ)_2_RbLa(NO_3_)_6_ (106 pC/N),^[^ ^6c^ ^]^ and [(CH_3_)_3_NCH_2_Br][MnBr_3_] (112 pC/N) ,^[^ ^19^ ^]^ some classical inorganic ferroelectrics like LiNbO_3_ (14 pC/N) ,^[^ ^20^ ^]^ and polymer ferroelectric PVDF (≈30 pC/N).^[^ ^21^ ^]^ We have measured the quasi‐static piezoelectric coefficients (*d*_33_) of a dozen polycrystalline pellets of [3.2.1‐abco]ReO_4_ at room temperature after poling processing, which is in the range of 107–118 pC/N with the average value of 114 pC/N, demonstrating satisfactory reproducibility of high piezoelectric response. Together with the easy fabrication process and ready availability of polycrystalline samples for various desired shapes, these attributes make [3.2.1‐abco]ReO_4_ highly attractive for a diverse range of piezoelectric‐related applications.

We further investigated the ferroelectric performance in bulk polycrystalline forms of [3.2.1‐abco]ReO_4_. The well‐shaped polarization−electric field (*P−E*) hysteresis loops in the temperature range of 300−338 K offer decisive proof for ferroelectricity, with remnant polarization (*P*_r_) of 4.3−5.1 µC cm^−2^ (Figure 3b). The obtained *P−E* hysteresis loops retain good rectangularity, indicating the robust ferroelectricity in its polycrystalline tablet. The *P*_r_ value at room temperature (4.7 µC cm^−2^ at 300 K) is higher than the typical molecular ferroelectric single‐crystal triglycine sulfate (2.8 µC cm^−2^),^[^ ^1^ ^]^ and comparable to those of other polycrystalline molecular ferroelectric such as [(CH_3_)_4_N][FeBrCl_3_] (4.5 µC cm^−2^) and [3.2.1‐dabco]BF_4_ (5.5 µC cm^−2^).^[^ ^13^, ^15^ ^]^ Notably, the coercive field (*E*_c_) shows a decrease with increasing temperature and obtains a small *E*_c_ of 7.6 kV cm^−1^ at 338 K, which should be due to the weakening of the coupling stabilizing effect between domains.^[^ ^22^ ^]^ Such good ferroelectric performances with small *E*_c_ values shown from polycrystalline pellets are expected to enable low voltage polarization switching of thin films of [3.2.1‐abco]ReO_4_. Under an AC frequency of 250 Hz, we observed well‐defined polarization−voltage hysteresis loops with a low coercive voltage of 2.2 V and a *P*_r_ value of 4.6 µC cm^−2^ (Figure 3c). Moreover, the thin film supports high‐frequency operation, and its ferroelectric polarization can be switched at frequencies even up to 200 kHz, with increased coercive voltages (< 10 V) (Figure 3d). The low coercive voltage in combination with the high‐frequency performance makes [3.2.1‐abco]ReO_4_ promisingly practical potential in low‐voltage‐driven electronic devices. Such a low coercive field can be attributed to the easy molecular reorientation during polarization switching, which is caused by the rotating disordered state in a certain orientation but in a polar alignment in the 3*m* phase. Additionally, we also investigated the elastic modulus (*E*) and hardness (*H*) of [3.2.1‐abco]ReO_4_ thin film by nanoindentation characterization, which is widely used to characterize the mechanical properties of molecular crystals.^[^ ^10^, ^23^ ^]^ From the fitted load–displacement curves based on the Oliver–Pharr model,^[^ ^24^ ^]^ we obtained the *E* and *H* of [3.2.1‐abco]ReO_4_ to be 6.93 GPa and 57.3 MPa, respectively (Figure S7, Supporting Information). The [3.2.1‐abco]ReO_4_ exhibit smaller *E* and *H* than those of the parent compound [2.2.1‐abch]ReO_4_ with *E* and *H* of 9.14 GPa and 75.5 MPa, respectively (Figure S8, Supporting Information), indicating the easier molecular deformation of the ring‐enlarged compound. The favorable mechanical properties of [3.2.1‐abco]ReO_4_ indicate good softness, ductility, and flexibility to have great application prospect in flexible devices.

For ferroelectric single crystals, the ideal single domain processing can make the spontaneous polarization of all parts of the entire crystal align in the direction of the electric field, that is, the residual polarization equals the spontaneous polarization. However, for ferroelectric ceramics, this is not the case because the spatial orientation of each crystal grain is inconsistent. Under the action of a sufficiently strong electric field, the spontaneous polarization of each grain takes the possible orientation closest to the direction of the electric field. Assuming a spherical surface and translating the spontaneous polarization vector of each grain so that the starting point is located at the center of the sphere. Before polarization processing, each vector endpoint is evenly distributed on the spherical surface. After polarization treatment, they are concentrated on a spherical cap with the electric field direction as the axis of symmetry. Assume that the angle between the spontaneous polarization of any grain and the electric field direction is *θ*, the polarization of the ceramic along the direction of the electric field can be obtained by the following formula,

(1) $$P_{e}=P_{s}\;\bar{cos\theta }$$

(2) $$\bar{cos\theta }=\frac{\int cos\theta dA}{\int dA}\;=\frac{\int cos\theta sin\theta d\theta }{\int sin\theta d\theta }\;$$

where *dA* is the assumed spherical area element, *P*_s_ is the spontaneous polarization, and *P*_e_ is the polarization of ferroelectric ceramic along the electric field direction.

**Figure 4:**
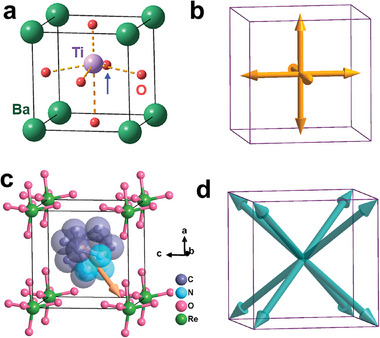
a,c) Packing view of unit cell of crystal structures for a) BaTiO_3_ in polar 4 mm phase and c) [3.2.1‐abco]ReO_4_ in polar 3*m* phase. b,d) The scheme drawing of possible equivalent polarization directions in the 4 mm and 3*m* phase respectively.

At room temperature, inorganic BaTiO_3_ is in a tetragonal 4 *mm* ferroelectric phase (**Figure** **4**a), and there are six possible orientations for spontaneous polarization, i.e., [001] (Figure 4b); [3.2.1‐abco]ReO_4_ is in a trigonal 3*m* ferroelectric phase at room temperature and has eight possible orientations for spontaneous polarization, i.e., [111] (Figure 4c,d). According to the above formula, regarding the cubic *m* $\bar{3}$ *m* paraelectric phase, the tetragonal 4 mm BaTiO_3_ ceramics after polarization treatment have a *P*_e_ = 0.83*P*_s_; For [3.2.1‐abco]ReO_4_ with trigonal 3*m* ferroelectric phase, *P*_e_ = 0.87*P*_s_. Therefore, like BaTiO_3_, the ceramic‐like [3.2.1‐abco]ReO_4_ in its powder compaction form can maintain a large polarization with a slight attenuation compared to single crystals. In combination with multiaxial ferroelectricity and low *E*_c_ induced by the rotating orientational disorder of cations in the 3*m* polar phase, the bulk polycrystalline pellet of [3.2.1‐abco]ReO_4_ can exhibit macroscopic ferroelectric performance. Such multiaxial ferroelectric characteristics, easily reoriented polarization, and large residual polarization promote the large piezoelectric response of plastic [3.2.1‐abco]ReO_4_ in powder compaction form.

**Figure 5:**
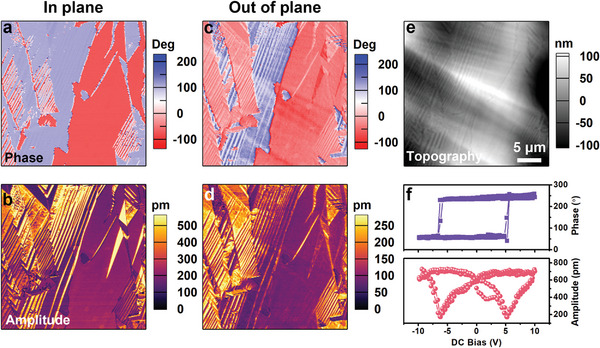
As‐grown ferroelectric domain structures of [3.2.1‐abco]ReO_4_ are shown in IP PFM phase a) and amplitude b) images, and in OP PFM phase c) and amplitude d) images. e) The topography image of the same region. f) PFM switching spectroscopy showing piezoelectric phase (up) and amplitude (down) hysteresis loops.

Piezoresponse force microscopy (PFM) is a mainstream technique for characterizing ferroelectric behaviors, which images the ferroelectric domains by mapping the piezoelectric strain in the sample surface generated under AC bias. Surface scanning while detecting vertical (out‐of‐plane, OP) surface vibration can yield a domain pattern that reflects the OP component of the polarization. Lateral PFM detects the in‐plane (IP) component of the polarization. The PFM images representing the IP and OP phase and amplitude signals of [3.2.1‐abco]ReO_4_ thin film at room temperature are shown in **Figure** **5**a–d, along with a topographic image acquired from the same measurement region (Figure 5e). Both IP (Figure 5a,b) and OP (Figure 5c,d) PFM images exhibited spontaneous polarization variations, indicating that the local neighboring domains show different directions of spontaneous polarizations and respond differently to the AC field. We then carried out PFM switching spectroscopy measurements on a single point of the thin film crystallite to study polarization switching (Figure 5f). Two cycles of DC bias up to ±10 V were applied to the sample via the conductive PFM tip. Simultaneously, a 2 V AC bias was applied to record the piezoelectric phase and amplitude. From the phase hysteresis loops, we observed that the 180˚ phase reversal occurs at −6.2 and +5.1 V on the negative and positive sides, respectively. Meanwhile, the butterfly‐shaped amplitude loops were obtained. The amplitude minimum corresponds to the coercive voltages, that is −6.2 and +5.1 V, coinciding well with the results from the phase loops. The square‐shaped phase loops and butterfly‐shaped amplitude loops provide robust evidence of electric field‐induced polarization switching in [3.2.1‐abco]ReO_4_.

**Figure 6:**
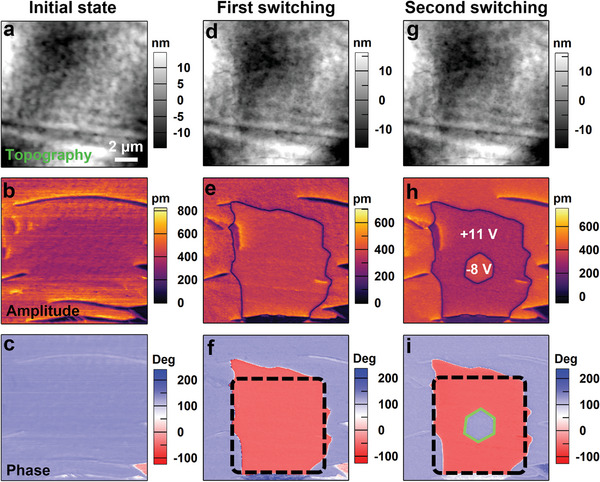
Topographic (up row), OP PFM amplitude (middle row), and phase (bottom row) images of the thin film surface for [3.2.1‐abco]ReO_4_. a–c) Initial state. d–f) After the first switching produced by scanning with a tip voltage of +11 V. g–i) After the second switching produced by scanning with an opposite tip voltage of −8 V.

Further, an attempt was made to visualize the polarization switching process through the domain writing experiment. **Figure** **6**a–c are topography, OP PFM amplitude, and phase images of the original sample, respectively. The selected region shows an up‐polarized pristine state. The polarization can be switched by applying a DC tip voltage via the PFM tip. Shown in Figure 6d–f are topography, OP PFM amplitude, and phase images after applying a DC tip voltage of +11 V on the central squared region. In this process, the applied voltage is nearly twice as large than the coercive voltage, causing the new domain to diffuse considerably. Next, an opposite tip voltage of −8 V was applied to back switch the newly generated domain, as shown in Figure 6g–i. Since the applied voltage is a little larger than the coercive voltage, the domain diffusion is according to the trigonal point group symmetry of phase II, resulting in a hexagonal shape.

**Figure 7:**
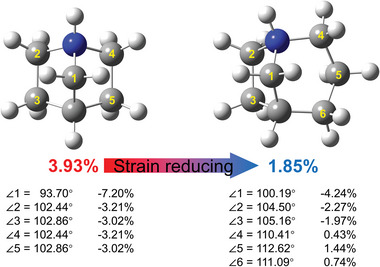
Molecular strain reducing from 3.93% in [2.2.1‐abch]^+^ cation to 1.85% in [3.2.1‐abco]^+^ cation. The angle represents the bond angle of each methylene (‐CH_2_‐) in the ring, and the following percentage represents the deformation of the carbon bond.

To investigate the enhancement of piezoelectric response in [3.2.1‐abco]ReO_4_ after ring enlargement. We quantitatively assess the magnitude of molecular strains of [3.2.1‐abco]^+^ cation in [3.2.1‐abco]ReO_4_ and [2.2.1‐abch]^+^ cation in [2.2.1‐abch]ReO_4_. We first optimize the molecular geometry at b3lyp‐D3(BJ)/6‐31G(d) level. The bond angle of each methylene can be directly measured from the two obtained stable molecular cations (**Figure** **7**). Then, half of the difference between the bond angle of the cycloalkanes and the normal bond angle (109°28') divided by the normal bond angle is defined as the degree of deformation of the carbon bond. Therein, the methylene group with label 1 in [2.2.1‐abch]^+^ cation has the largest degree of deformation (−7.20%), which is reduced into −4.24% in [3.2.1‐abco]^+^ cation. The two ethylene groups (labeled as 23 and 45, respectively) are symmetric about the mirror, with the degree of methylene group deformation of −3.21% and −3.02% in [2.2.1‐abch]^+^ cation, which are slightly reduced into −2.27% and −1.97% in [3.2.1‐abco]^+^ cation. And finally, the deformation of the carbon bond has experienced great change from ≈3% to 0.43%–1.44% (labeled as 4, 5 and 6) by adding a methylene. Based on all the above data, we define the average absolute value of the degree of deformation of each methylene as the molecular strain. Therefore, the total molecular strain is reduced from 3.93% ([2.2.1‐abch]^+^) to 1.85% ([3.2.1‐abco]^+^) by ring enlargement. The reduction of molecular strain enables the easier deformation of polar [3.2.1‐abco]^+^ cation. When the external stress is applied to the 3*m* ferroelectric phase of [3.2.1‐abco]ReO_4_ where [3.2.1‐abco]^+^ cation shows rotating orientational disorder, the easier molecular deformation induces a larger change of the polarization and the higher piezoelectric response.

## 3 Conclusion

In summary, through ring enlargement, we have synthesized a polycrystalline molecular ferroelectric 1‐azabicyclo[3.2.1]octonium perrhenate ([3.2.1‐abco]ReO_4_) with a large *d*_33_ up to 118 pC/N in the polycrystalline pellet form, which is higher than that of the parent 1‐azabicyclo[2.2.1]heptanium perrhenate (90 pC/N) and those of most molecular ferroelectrics in polycrystalline or even single crystal forms. The multipolar axis characteristic and plastic malleability allows [3.2.1‐abco]ReO_4_ to display macroscopic ferroelectricity in both polycrystalline pellet and thin‐film forms, which enable low‐voltage operations and high‐frequency performance of polarization switching. Combined with the room‐temperature fabrication of either aqueous method for single crystals or simple cold‐pressing method for polycrystalline forms, the [3.2.1‐abco]ReO_4_ manifests promising prospects in a wide range of applications such as soft piezoelectric devices, sensor elements, flexible and biomedical electronics, *etc*. The emergence of such type of molecular polycrystalline ferroelectrics has brought unprecedented feasibility for commercial applications, and with continuous in‐depth research, to finally launch a shaking challenge to the current preeminence of inorganic ferroelectric ceramics.

## 4 Experimental Section

### Crystal Growth, Thin‐Film and Polycrystalline Pellet Preparation

1‐Azabicyclo[3.2.1]octane was synthesized according to the reported literature.^[^ ^25^ ^]^ [3.2.1‐abco]ReO_4_ was easily obtained as colorless block single crystals through slow evaporation of methanol solution consisting of equimolar 1‐azabicyclo[3.2.1]octane and perrhenic acid. The synthesis route (Figure S9, Supporting Information) and detailed synthesis recipe for 1‐azabicyclo[3.2.1]octane and [3.2.1‐abco]ReO_4_ were described. The hydrogen atoms and the structure of the [3.2.1‐abco]ReO_4_ were confirmed by ^1^H NMR (nuclear magnetic resonance) (Figures S10 and S11, Supporting Information) and the ^13^C NMR (Figure S12, Supporting Information). The single peak for protonated ‐NH^+^ of [3.2.1‐abco]ReO_4_ was observed at *δ* = 9.55 ppm, where DMSO‐*d*_6_ was used as the solvent (Figure S10, Supporting Information). This chemical shift of the protonated ‐NH^+^ disappeared when D_2_O was used as the solvent due to the deuterium effect of active hydrogen (Figure S11, Supporting Information). Apparently, the chemical shift of the H atom at *δ* = 9.55 ppm confirmed that the proton transferred from HReO_4_ to the 1‐azabicyclo[3.2.1]octane due to the acidification. It was also recorded the infrared (IR) spectrum of [3.2.1‐abco]ReO_4_ (Figure S13, Supporting Information), which shows the existence of typical strong stretching vibration peaks of Re=O bonds at 908 cm^−1^ and the characteristic peak of the N‐H stretching vibration at 3147 cm^−1^. The precursor solution of [3.2.1‐abco]ReO_4_ was prepared by dissolving 20 mg of the crystals in 0.2 mL aqueous solution. A 20 µL portion of the precursor solution was spread on a clean indium tin oxide (ITO) glass substrate. High‐quality thin films with thicknesses of ≈500 nm was formed after slow solvent evaporation at 300 K. The polycrystalline pellet with thickness of ≈0.2 mm was prepared by cold‐pressing of ground crystal powders in a cylinder mold.

### X‐Ray Diffraction Measurement

The single‐crystal diffraction data of [3.2.1‐abco]ReO_4_ at 373, 300, 253, and 100 K by using Rigaku Oxford diffractometer with Cu‐K*α* radiation (*λ* = 1.54178 Å) was obtained. The structures by direct methods and refined them by the full‐matrix method based on the SHELXTL software package was solved. The C, N, Re, and O atoms were refined anisotropically. The X‐ray crystallographic structures have been deposited at the Cambridge Crystallographic Data Centre (deposition numbers CCDC 2252194–2252197) and can be obtained free of charge from the CCDC via www.ccdc.cam.ac.uk/getstructures. Powder X‐ray diffraction (PXRD) patterns were collected by using a Rigaku Smartlab X‐ray diffraction system with Cu K*α* radiation. For PXRD patterns, the recorded 2*θ* angles range is 5–50°, and the step size was 0.02°.

### DSC, SHG, and Dielectric Measurements

The DSC curves on a PerkinElmer DSC 6000 instrument under a nitrogen atmosphere at a heating and cooling rate of 10 K min^−1^ was measured. The polycrystalline powder sample was placed in aluminum crucibles. It was performed temperature‐dependent SHG intensity on Ins 1 210 058, INSTEC Instruments using an unexpanded laser beam with low divergence (pulsed Nd: YAG at a wavelength of 1064 nm, 5 ns pulse duration, 1.6 MW peak power, 10 Hz repetition rate), and a Linkam LTS420 thermal stage was equipped to control the temperature. The laser was Vibrant 355 II, OPOTEK. The complex dielectric permittivity curves were measured on an automatic impedance Tonghui 2828 analyzer at the frequency of 1 MHz with the AC (Alternating Current) voltage of 1.0 V. For the dielectric measurements, the polycrystalline sample into powder and pressed it into a thin plate was first grounded. Then the conductive silver glue on both top and bottom plate surfaces as the electrodes of the sample sheet was deposited. Finally, silver conducting glue to stick the copper wire on it for connection with the six‐hole socket to form a capacitor was used.

### Ferroelectric Hysteresis Loop

For the polycrystalline pellet, the sample was pasted with conductive silver glue on both faces to form an Ag/[3.2.1‐abco]ReO_4_/Ag architecture. For the thin film samples, the liquid GaIn eutectic was dropped on the sample as the top electrode to form an ITO/[3.2.1‐abco]ReO_4_/GaIn capacitor architecture. The *P–E* measurements were performed on a probe station equipped with a Precision Premier II Ferroelectric Tester (Radiant Technologies), and typical triangular wave was applied with different combinations of voltages and periods.

### PFM Characterization

PFM measurements were carried out on a commercial scanning probe microscope (Oxford instrument, Cypher ES) instrument to characterize the surface morphology, domain structure, PFM switching spectroscopy, and domain switching. Conductive Pt/Ir‐coated silicon probes (EFM, Nanoworld) with a nominal spring constant of ≈2.8 nN/nm and a free‐air resonance frequency of ≈75 kHz were used. To enhance the signal‐to‐noise ratio, the resonance‐enhanced mode was used, with the tip driven with an AC voltage of 5 V at the vertical contact resonance (≈300 kHz) and an AC voltage of 2 V at the lateral contact resonance (≈620 kHz).

### Nanoindentation Method

A Bruker Hysitron TI Premier nanoindenter with a Berkovich probe (tip radius of 150 nm) was utilized to perform nanoindentaion test to reveal elastic modulus (*E*) and hardness (*H*). The sample was firmly fixed onto the sample stage by cyanoacrylate glue. Standard load function was set with peak load of 200 µN during test. The *H* and *E* values were obtained by fitting the load‐displacement curve based on the Oliver‐Pharr method using the pre‐installed software on the device. The load‐displacement data were collected from four different positions with a distance of 10 µm to minimize test error.

### Poling Method

The [3.2.1‐abco]ReO_4_ polycrystalline pellet was treated with an electric field of 10 kV cm^−1^ in dimethyl silicone at the temperature of 323 K for 30 min.

## Conflict of Interest

The authors declare no conflict of interest.

## Supporting information

Supporting Information

Supporting Information

## Data Availability

Research data are not shared.

## References

[1] M. E. Lines , A. M. Glass , Principles and applications of ferroelectrics and related materials, Oxford University Press, Oxford, 2001.

[2] a) J. F. Scott , Science 2007, 315, 954; 17303745 10.1126/science.1129564

[3] a) C. A. Randall , N. Kim , J. P. Kucera , W. W. Cao , T. R. Shrout , J. Am. Ceram. Soc. 1998, 81, 677;

[4] L. B. Kong , T. Zhang , J. Ma , F. Boey , Prog. Mater. Sci. 2008, 53, 207.

[5] a) S. Horiuchi , Y. Tokura , Nat. Mater. 2008, 7, 357; 18432209 10.1038/nmat2137

[6] a) Y. Hu , L. You , B. Xu , T. Li , S. A. Morris , Y. Li , Y. Zhang , X. Wang , P. S. Lee , H. J. Fan , J. Wang , Nat. Mater. 2021, 20, 612; 33432147 10.1038/s41563-020-00875-3

[7] a) S. Horiuchi , Y. Tokunaga , G. Giovannetti , S. Picozzi , H. Itoh , R. Shimano , R. Kumai , Y. Tokura , Nature 2010, 463, 789; 20148035 10.1038/nature08731

[8] a) D.‐W. Fu , H.‐L. Cai , Y. Liu , Q. Ye , W. Zhang , Y. Zhang , X.‐Y. Chen , G. Giovannetti , M. Capone , J. Li , R.‐G. Xiong , Science 2013, 339, 425; 23349285 10.1126/science.1229675

[9] S. Horiuchi , F. Ishii , R. Kumai , Y. Okimoto , H. Tachibana , N. Nagaosa , Y. Tokura , Nat. Mater. 2005, 4, 163. 15665837 10.1038/nmat1298

[10] a) S. Das , A. Mondal , C. M. Reddy , Chem. Soc. Rev. 2020, 49, 8878; 33185234 10.1039/d0cs00475h

[11] a) J. Harada , T. Shimojo , H. Oyamaguchi , H. Hasegawa , Y. Takahashi , K. Satomi , Y. Suzuki , J. Kawamata , T. Inabe , Nat. Chem. 2016, 8, 946; 27657871 10.1038/nchem.2567

[12] a) J. Harada , APL Mater. 2021, 9, 020901;

[13] a) A. Mondal , B. Bhattacharya , S. Das , S. Bhunia , R. Chowdhury , S. Dey , C. M. Reddy , Angew. Chem., Int. Ed. 2020, 59, 10971; 10.1002/anie.202001060 32087039

[14] S. Bhunia , S. Chandel , S. K. Karan , S. Dey , A. Tiwari , S. Das , N. Kumar , R. Chowdhury , S. Mondal , I. Ghosh , A. Mondal , B. B. Khatua , N. Ghosh , C. M. Reddy , Science 2021, 373, 321. 34437150 10.1126/science.abg3886

[15] a) Q. Pan , Z. B. Liu , H. Y. Zhang , W. Y. Zhang , Y. Y. Tang , Y. M. You , P. F. Li , W. Q. Liao , P. P. Shi , R. W. Ma , R. Y. Wei , R. G. Xiong , Adv. Mater. 2017, 29, 1700831; 10.1002/adma.201700831 28585230

[16] X.‐G. Chen , Y.‐Y. Tang , H.‐P. Lv , X.‐J. Song , H. Peng , H. Yu , W.‐Q. Liao , Y.‐M. You , R.‐G. Xiong , J. Am. Chem. Soc. 2023, 145, 1936. 36637030 10.1021/jacs.2c12306

[17] Y. Zhang , Y. Liu , H.‐Y. Ye , D.‐W. Fu , W. Gao , H. Ma , Z. Liu , Y. Liu , W. Zhang , J. Li , G.‐L. Yuan , R.‐G. Xiong , Angew. Chem., Int. Ed. 2014, 53, 5064. 10.1002/anie.201400348 24692257

[18] H. Zhang , Z.‐K. Xu , Z.‐X. Wang , H. Yu , H.‐P. Lv , P.‐F. Li , W.‐Q. Liao , R.‐G. Xiong , J. Am. Chem. Soc. 2023, 145, 4892. 36795554 10.1021/jacs.3c00646

[19] W. Q. Liao , Y. Y. Tang , P. F. Li , Y. M. You , R. G. Xiong , J. Am. Chem. Soc. 2017, 139, 18071. 29144132 10.1021/jacs.7b10449

[20] A. Warner , M. Onoe , G. Coquin , J. Acoust. Soc. Am. 1967, 42, 1223.

[21] Q. Li , Q. Wang , Macromol. Chem. Phys. 2016, 217, 1228.

[22] L. Jin , F. Li , S. Zhang , J. Am. Ceram. Soc. 2014, 97, 1.

[23] a) S. Varughese , M. S. R. N. Kiran , U. Ramamurty , G. R. Desiraju , Angew. Chem., Int. Ed. 2013, 52, 2701; 10.1002/anie.201205002 23315913

[24] W. C. Oliver , G. M. Pharr , J. Mater. Res. 1992, 7, 1564.

[25] A. D. Yanina , Zh. Obshch. Khim. 1959, 29, 485.

