# Supplementary material for: The First Ring Enlargement Induced Large Piezoelectric Response in a Polycrystalline Molecular Ferroelectric
Source: Adv Sci (Weinh). 2023 Jun 16;10(24):2302426. doi: 10.1002/advs.202302426 (PMC10460893; doi:10.1002/advs.202302426)
Supplement: Supplementary file 1 — Supporting Information [file ADVS-10-2302426-s001.pdf]

ADVANCED SCIENCE
Open Access

Supporting Information

for *Adv. Sci.*, DOI 10.1002/advs.202302426

The First Ring Enlargement Induced Large Piezoelectric Response in a Polycrystalline Molecular Ferroelectric

Yong Ai, Peng-Fei Li, Xiao-Gang Chen, Hui-Peng Lv, Yan-Ran Weng, Yu Shi, Feng Zhou, Ren-Gen Xiong and Wei-Qiang Liao**

Supporting Information

The First Ring Enlargement Induced Large Piezoelectric Response in a Polycrystalline Molecular Ferroelectric

Yong Ai, Peng-Fei Li, Xiao-Gang Chen, Hui-Peng Lv, Yan-Ran Weng, Yu Shi, Feng Zhou, Ren-Gen Xiong* and Wei-Qiang Liao*

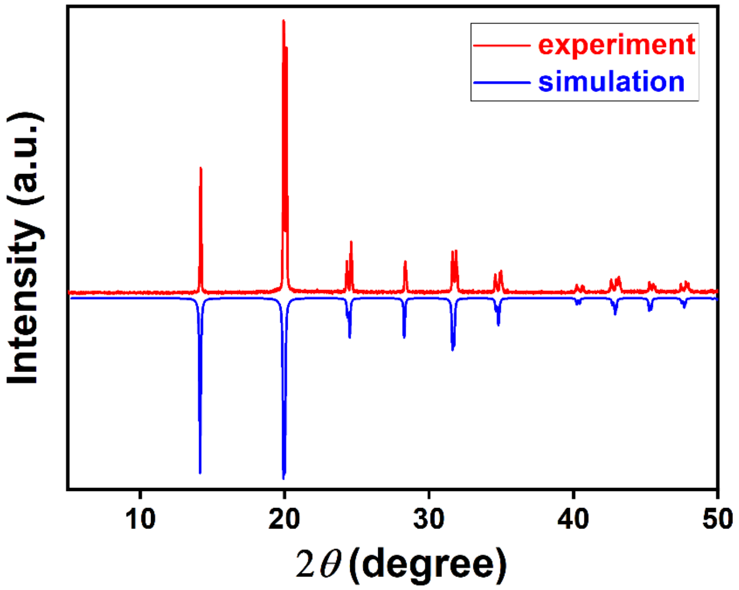

Figure S1. The experimental PXRD patterns of [3.2.1-abco]ReO_4 at room temperature, match well with the simulated PXRD patterns.

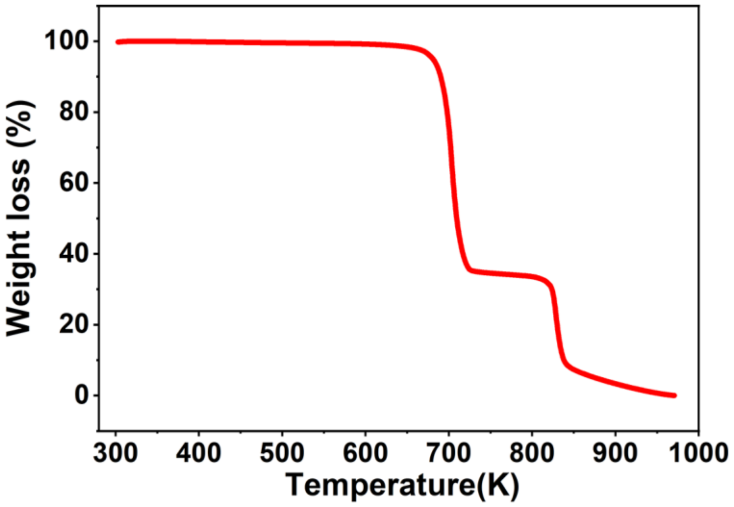

Figure S2. Thermogravimetric analysis (TGA) of [3.2.1-abco]ReO_4.

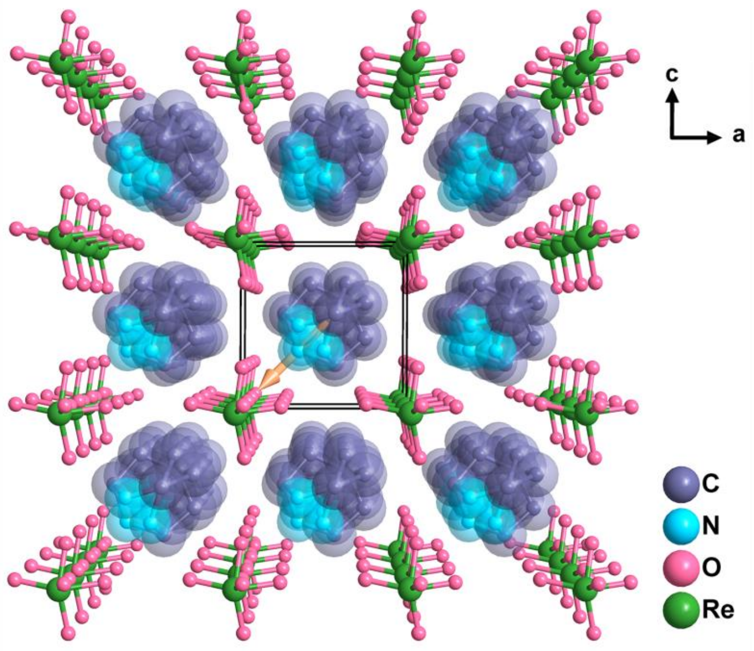

Figure S3. Packing view of crystal structure of [3.2.1-abco]ReO_4 at 300 K, in which all the [3.2.1-abco]$^+$ cations are aligned in a head-to-tail manner along the longer body diagonal direction of the crystal cell.

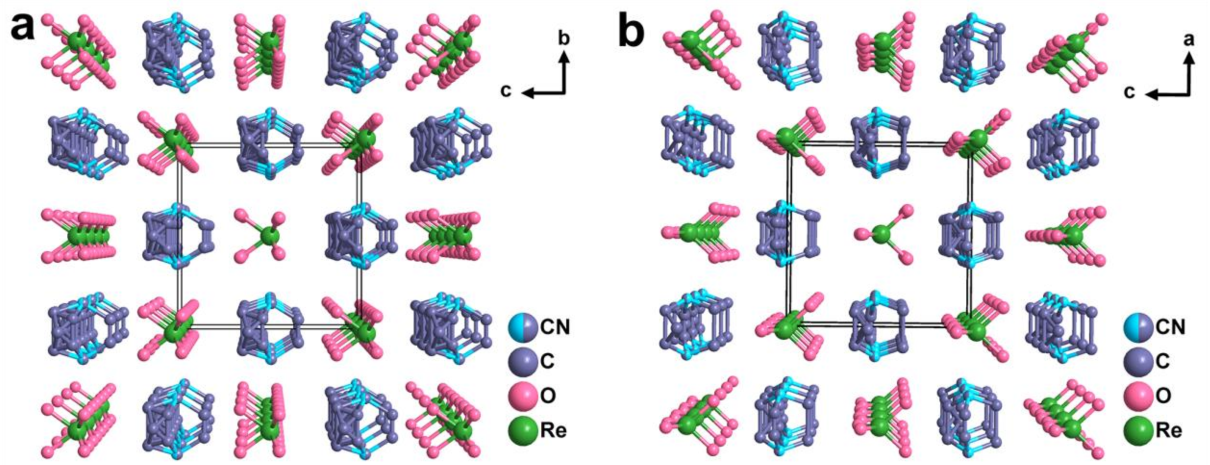

Figure S4. Packing view of crystal structure of [3.2.1-abco]ReO_4 at (a) 253 K and (b) 100 K.

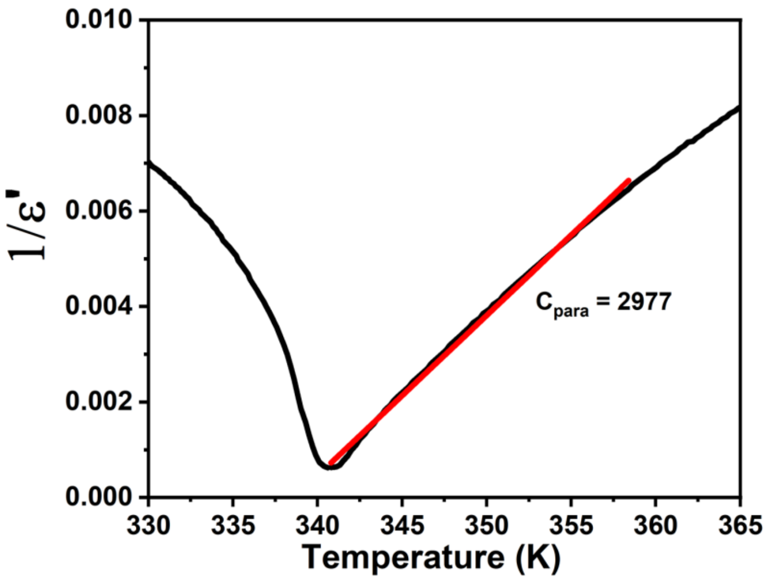

Figure S5. A linear fit to the Curie−Weiss law using ε' values at 1 MHz.

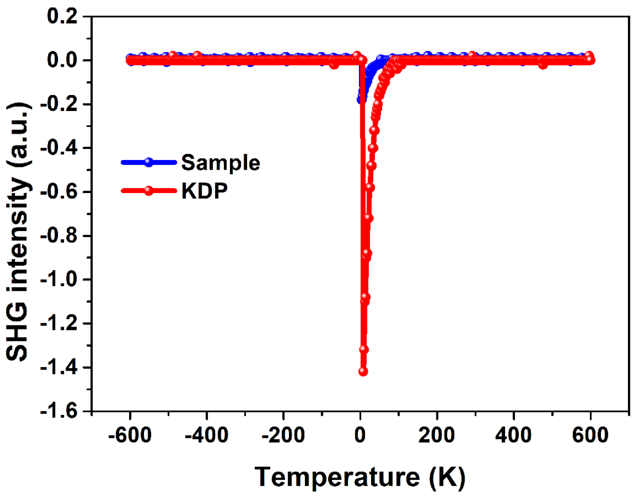

Figure S6. The SHG intensity of [3.2.1-abco]ReO_4 compared to that of KDP at 300 K.

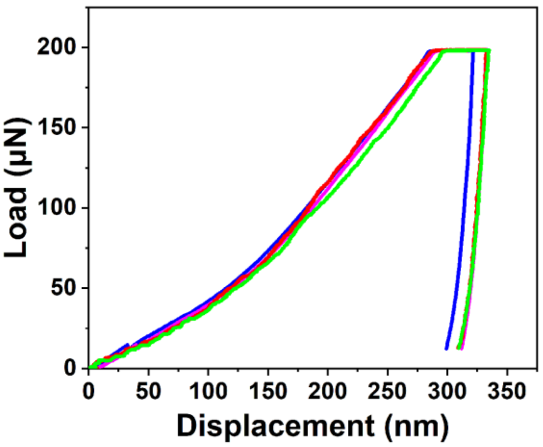

Figure S7. Load–displacement curves of the [3.2.1-abco]ReO_4 tested by nanoindentation revealing elastic modulus and hardness.

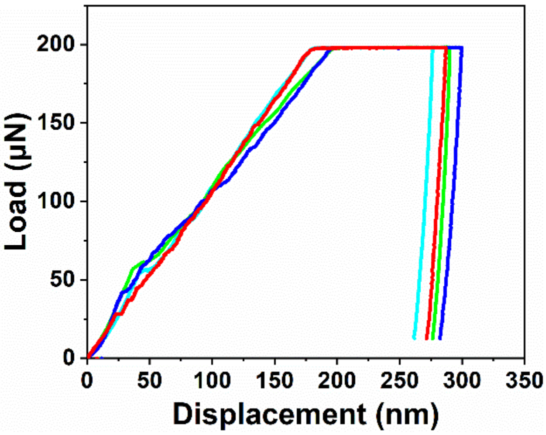

Figure S8. Load–displacement curves of the [2.2.1-abch]ReO_4 tested by nanoindentation technique.

The detailed synthetic procedure for 1-azabicyclo[3.2.1]octane and [3.2.1-abco]ReO_4:

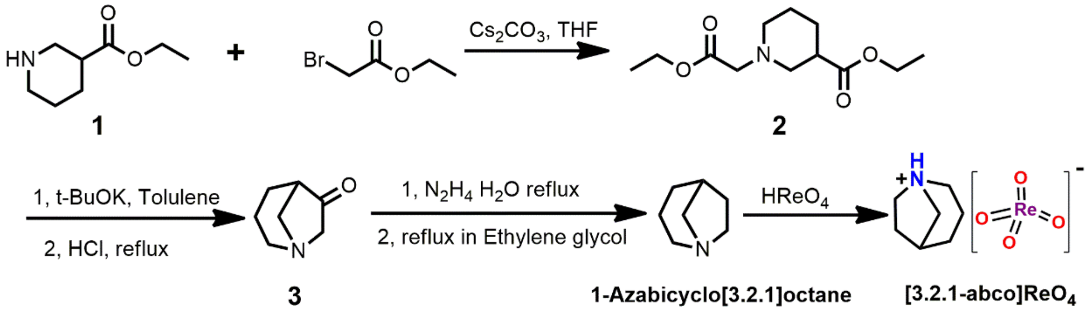

Figure S9. Synthesis route of 1-azabicyclo[3.2.1]octane and [3.2.1-abco]ReO_4.

Materials. Ethyl piperidine-3-carboxylate (compound **1**), ethyl bromoacetate, Cs_2CO_3, *t*-BuOK, $HReO_4$ (80% in water) and Na_2SO_4 were purchased from Macklin company and used as-received. Hydrochloric acid (HCl) was purchased from Adamas. Hydrazine hydrate were purchased from Aladdin company. Tetrahydrofuran (THF) and toluene were purchased from Adamas and used as solvent.

Compound 2. To a THF solution in a round bottom flask was added ethyl piperidine-3-carboxylate (1) (10.0 g, 63.6 mmol), Cs_2CO_3 (22.8 g, 70.0 mmol). Ethyl bromoacetate (11.7g, 70.0mmol) was dropped into the solution and the mixture was refluxed for 4h. The filtrate was collected and the THF solvent was removed under vacuum. Compound 2 was obtained as light-yellow oil and used for further step without any purification. GC-MS m/z (M^+): 243.15.

Compound 3. To an anhydrous toluene solution was dissolved Compound 2 (10.0 g, 41.1 mmol) and added *t*-BuOK (6.9 g, 61.7 mmol). The mixture was refluxed at 115°C for 4h. The mixture solution was cooled to room temperature, extracted with concentrated HCl (100 ml × 3). The aqueous phase was collected and refluxed at 110°C overnight. Then the saturated NaOH

solution was added under ice bath until the pH > 8. The residue was extracted with ethyl acetate (100 mL × 5). The organic layer was collected, dried over with Na_2SO_4, filtered, and concentrated. The crude product of compound 3 was obtained as a light brown solid. GC-MS m/z (M^+): 125.10.

1-Azabicyclo[3.2.1]octane. Compound 3 (10.0 g, 80mmol) was dissolved in 30 ml hydrazine hydrate (98%) and refluxed at 120 °C for 8h. Then KOH (9.0 g, 160 mmol) and ethylene glycol (40 ml) was added. The mixture was refluxed at 160 °C overnight. The ethylene glycol was removed by reduced-pressure distillation. The residue was dissolved in 200 ml water and extracted with ethyl acetate (100 mL × 5). The organic layer was collected, dried over with Na_2SO_4, filtered, and concentrated. GC-MS shows the m/z (M^+) of 1-Azabicyclo[3.2.1]octane was 111.15.

[3.2.1-abco]ReO4. To the methanol solution of 1-azabicyclo[3.2.1]octane added $HReO_4$ (80% in water) until pH < 7. The precipitation of [3.2.1-abco]ReO_4 was obtained immediately as white solid and purified through recrystallization in methanol solution. ^{1}H NMR (300 MHz, D_2O). δ = 3.58 – 3.19 (m, 6H), 2.71 (s, 1H), 2.24 (ddd, J = 13.7, 11.7, 6.9, 1H), 2.13 – 1.92 (m, 2H), 1.91 – 1.63 (m, 3H). ^{13}C NMR (75 MHz, D_2O) δ = 58.92, 53.33, 50.67, 33.36, 27.69, 26.74, 16.26.

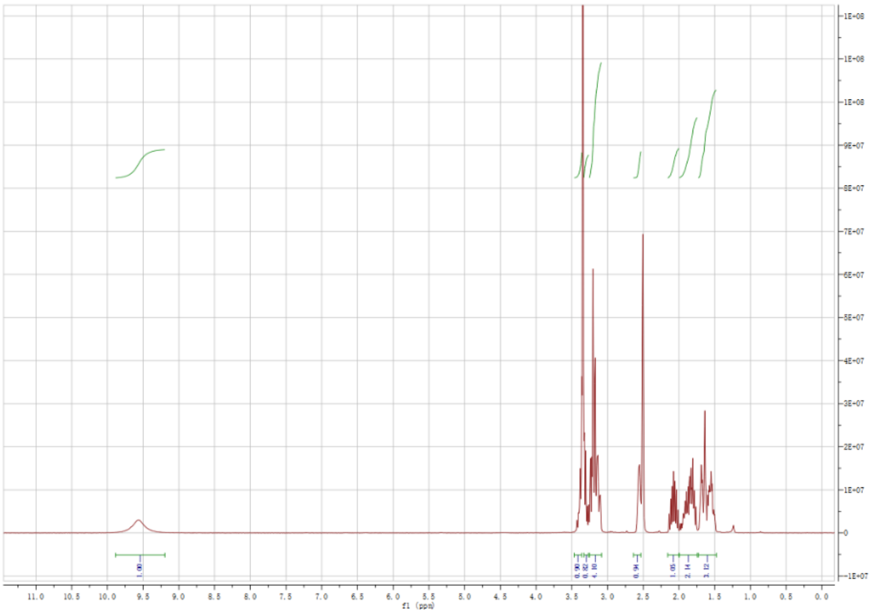

Figure S10. Image of ^{1}HNMR (300 MHz) for [3.2.1-abco]ReO_4, DMSO-d_6 as solvent. Solvent peak at 2.5 ppm and water peak at 3.35 ppm partially merged with peaks of [3.2.1-abco]ReO_4. NMR spectrometer was produced by Bruker Switzerland AG.

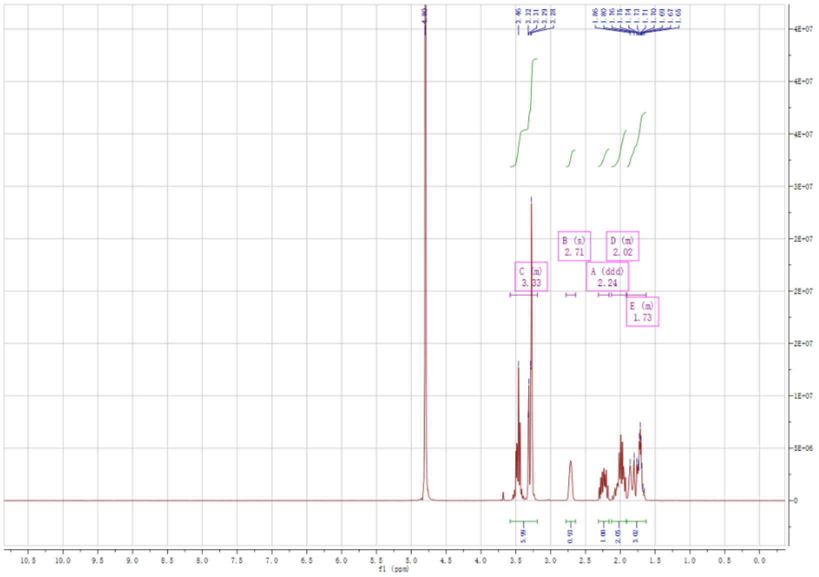

Figure S11. Image of ^{1}HNMR (300 MHz) for [3.2.1-abco]ReO_4, D_2O as solvent. NMR spectrometer was produced by Bruker Switzerland AG.

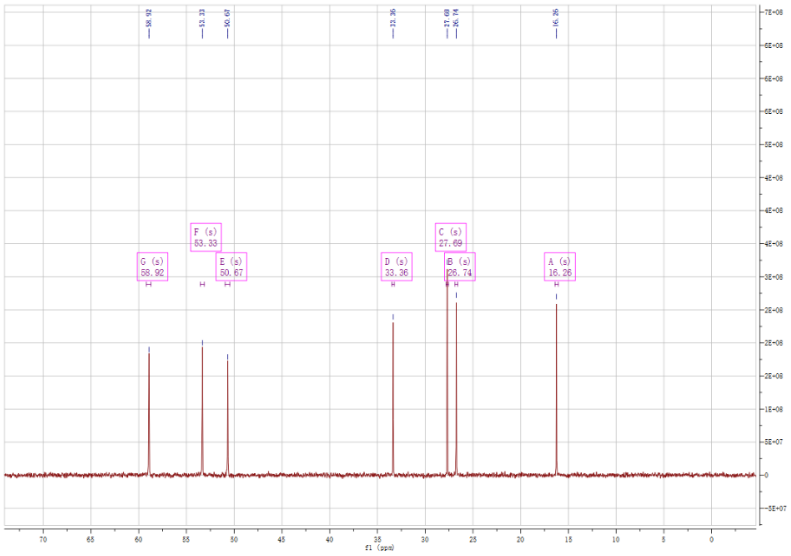

Figure S12. Image of ^{13}C NMR (75 MHz) for [3.2.1-abco]ReO_4, D_2O as solvent. NMR spectrometer was produced by Bruker Switzerland AG.

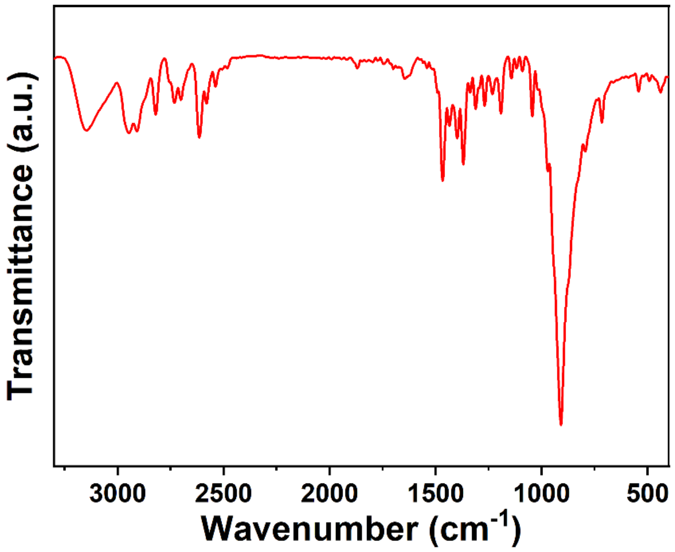

Figure S13. Infrared (IR) spectrum of [3.2.1-abco]ReO_4 recorded on a Shimadzu model INVENIO-R spectrometer at room temperature.

Table S1. Crystal data and structure refinement for [3.2.1-abco]ReO_4 at 373, 300, 253 and 100 K.

Temperature	373 K	300 K	253 K	100 K
System	cubic	trigonal	orthorhombic	orthorhombic
Space group	$Pm\bar{3}m$	$R3m$	$Amm2$	$Pmn2_1$
a (Å)	6.4362(3)	6.3552(8)	6.2218(6)	8.9677(6)
b (Å)	6.4362(3)	6.3552(8)	9.0504(6)	6.0634(6)
c (Å)	6.4362(3)	6.3552(8)	8.9667(7)	8.9688(6)
α (°)	90	89.73	90	90
β (°)	90	89.73	90	90
γ (°)	90	89.73	90	90
V (Å^3)	266.62(4)	256.67(10)	504.91(7)	487.68(7)
Z	1	1	2	2
R_{int}	0.0173	0.0242	0.0858	0.0498
R_1	0.0409	0.0778	0.0678	0.0891
wR_2	0.1211	0.2199	0.1860	0.2403
GOF	1.063	1.088	1.088	1.104

The piezoelectric constant matrix [d] for 3m point group can be expressed as:

$$\begin{bmatrix} 0 & 0 & 0 & 0 & d_{15} & -2d_{22} \\ -d_{22} & d_{22} & 0 & d_{15} & 0 & 0 \\ d_{31} & d_{31} & d_{33} & 0 & 0 & 0 \end{bmatrix} \quad \textbf{(eqn. S1)}$$
