# Supplementary material for: The First Ring Enlargement Induced Large Piezoelectric Response in a Polycrystalline Molecular Ferroelectric
Source: Adv Sci (Weinh). 2023 Jun 16;10(24):2302426. doi: 10.1002/advs.202302426 (PMC10460893; doi:10.1002/advs.202302426)

checkCIF/PLATON report

You have not supplied any structure factors. As a result the full set of tests cannot be run.

THIS REPORT IS FOR GUIDANCE ONLY. IF USED AS PART OF A REVIEW PROCEDURE FOR PUBLICATION, IT SHOULD NOT REPLACE THE EXPERTISE OF AN EXPERIENCED CRYSTALLOGRAPHIC REFEREE.

No syntax errors found. CIF dictionary Interpreting this report

Datablock: Pmn21

```
Bond precision:   C-C = 0.0173 A          Wavelength=1.54178

Cell:               a=8.9677(6)       b=6.0634(6)       c=8.9688(6)
                    alpha=90          beta=90           gamma=90
Temperature:        100 K

                 Calculated                   Reported
Volume           487.68(7)                    487.68(7)
Space group      P m n 21                     P m n 21
Hall group       P 2ac -2                     P 2ac -2
Moiety formula   C7 N, O4 Re                  C7 H0 N O4 Re
Sum formula      C7 N O4 Re                   C7 H0 N O4 Re
Mr               348.29                       348.28
Dx,g cm-3        2.372                        2.372
Z                2                            2
Mu (mm-1)        24.286                       24.286
F000             312.0                        312.0
F000'            302.41
h,k,lmax         11,7,11                      11,7,10
Nref             1070[ 570]                   668
Tmin,Tmax        0.083,0.088                  0.083,0.088
Tmin'            0.013

Correction method= # Reported T Limits: Tmin=0.083 Tmax=0.088
AbsCorr = MULTI-SCAN

Data completeness= 1.17/0.62       Theta(max)= 74.566

R(reflections)= 0.0891( 658)              wR2(reflections)=
                                          0.2403( 668)
S = 1.104                 Npar= 33
```

The following ALERTS were generated. Each ALERT has the format
test-name_ALERT_alert-type_alert-level.
Click on the hyperlinks for more details of the test.

Alert level C

```
STRVA01_ALERT_4_C          Flack test results are ambiguous.
           From the CIF: _refine_ls_abs_structure_Flack    0.560
           From the CIF: _refine_ls_abs_structure_Flack_su    0.110
PLAT029_ALERT_3_C _diffrn_measured_fraction_theta_full value Low .      0.970 Why?
PLAT218_ALERT_3_C Constrained U(ij) Components(s) for C3         .          2 Check
PLAT218_ALERT_3_C Constrained U(ij) Components(s) for C4         .          2 Check
PLAT218_ALERT_3_C Constrained U(ij) Components(s) for C2         .          2 Check
PLAT218_ALERT_3_C Constrained U(ij) Components(s) for N1         .          2 Check
PLAT218_ALERT_3_C Constrained U(ij) Components(s) for C1         .          2 Check
PLAT218_ALERT_3_C Constrained U(ij) Components(s) for O1         .          2 Check
PLAT260_ALERT_2_C Large Average Ueq of Residue Including        N1      0.130 Check
PLAT342_ALERT_3_C Low Bond Precision on  C-C Bonds ...............    0.01733 Ang.
PLAT907_ALERT_2_C Flack x > 0.5, Structure Needs to be Inverted? .       0.56 Check
```

Alert level G

```
CELLZ01_ALERT_1_G Difference between formula and atom_site contents detected.
CELLZ01_ALERT_1_G WARNING: H atoms missing from atom site list. Is this intentional?
           From the CIF: _cell_formula_units_Z    2
           From the CIF: _chemical_formula_sum  C7 H0 N O4 Re
           TEST: Compare cell contents of formula and atom_site data

           atom    Z*formula  cif sites diff
           C         14.00     14.00    0.00
           H          2.00      0.00    2.00
           N          2.00      2.00    0.00
           O          8.00      8.00    0.00
           Re         2.00      2.00    0.00
PLAT002_ALERT_2_G Number of Distance or Angle Restraints on AtSite          9 Note
PLAT003_ALERT_2_G Number of Uiso or Uij Restrained non-H Atoms ...         10 Report
PLAT012_ALERT_1_G N.O.K.   _shelx_res_checksum Found in CIF ......     Please Check
PLAT040_ALERT_1_G No H-atoms in this Carbon Containing Compound ..     Please Check
PLAT066_ALERT_1_G Predicted and Reported Tmin&Tmax Range Identical          ? Check
PLAT168_ALERT_4_G The CIF-Embedded .res File Contains EXYZ Records          1 Report
PLAT171_ALERT_4_G The CIF-Embedded .res File Contains EADP Records          3 Report
PLAT172_ALERT_4_G The CIF-Embedded .res File Contains DFIX Records          5 Report
PLAT177_ALERT_4_G The CIF-Embedded .res File Contains DELU Records          1 Report
PLAT178_ALERT_4_G The CIF-Embedded .res File Contains SIMU Records          1 Report
PLAT186_ALERT_4_G The CIF-Embedded .res File Contains ISOR Records          1 Report
PLAT188_ALERT_3_G A Non-default SIMU Restraint Value has been used     0.0100 Report
PLAT300_ALERT_4_G Atom Site Occupancy of N1        Constrained at        0.5 Check
PLAT300_ALERT_4_G Atom Site Occupancy of C1        Constrained at        0.5 Check
PLAT300_ALERT_4_G Atom Site Occupancy of C3        Constrained at        0.5 Check
PLAT301_ALERT_3_G Main Residue  Disorder ..............(Resd  1  )        38% Note
PLAT432_ALERT_2_G Short Inter X...Y Contact  C2        ..C5       .       2.85 Ang.
                                                    x,1+y,z  =      1_565 Check
PLAT773_ALERT_2_G Check long C-C Bond in CIF: C3       --C3               1.75 Ang.
PLAT860_ALERT_3_G Number of Least-Squares Restraints .............         76 Note
PLAT883_ALERT_1_G No Info/Value for _atom_sites_solution_primary .     Please Do !
PLAT941_ALERT_3_G Average HKL Measurement Multiplicity ...........        2.6 Low
```

```
PLAT965_ALERT_2_G The SHELXL WEIGHT Optimisation has not Converged     Please Check
```

```
   0 ALERT level A = Most likely a serious problem - resolve or explain
   0 ALERT level B = A potentially serious problem, consider carefully
  11 ALERT level C = Check. Ensure it is not caused by an omission or oversight
  24 ALERT level G = General information/check it is not something unexpected

   6 ALERT type 1 CIF construction/syntax error, inconsistent or missing data
   7 ALERT type 2 Indicator that the structure model may be wrong or deficient
  12 ALERT type 3 Indicator that the structure quality may be low
  10 ALERT type 4 Improvement, methodology, query or suggestion
   0 ALERT type 5 Informative message, check
```

Validation response form

Please find below a validation response form (VRF) that can be filled in and pasted into your CIF.

```
# start Validation Reply Form
_vrf_STRVA01_Pmn21
;
PROBLEM: Flack test results are ambiguous.
RESPONSE: ...
;
_vrf_PLAT029_Pmn21
;
PROBLEM: _diffrn_measured_fraction_theta_full value Low .      0.970 Why?
RESPONSE: ...
;
_vrf_PLAT218_Pmn21
;
PROBLEM: Constrained U(ij) Components(s) for C3         .          2 Check
RESPONSE: ...
;
_vrf_PLAT260_Pmn21
;
PROBLEM: Large Average Ueq of Residue Including        N1      0.130 Check
RESPONSE: ...
;
_vrf_PLAT342_Pmn21
;
PROBLEM: Low Bond Precision on  C-C Bonds ..............    0.01733 Ang.
RESPONSE: ...
;
_vrf_PLAT907_Pmn21
;
PROBLEM: Flack x > 0.5, Structure Needs to be Inverted? .       0.56 Check
RESPONSE: ...
;
# end Validation Reply Form
```

It is advisable to attempt to resolve as many as possible of the alerts in all categories. Often the minor alerts point to easily fixed oversights, errors and omissions in your CIF or refinement strategy, so attention to these fine details can be worthwhile. In order to resolve some of the more serious problems it may be necessary to carry out additional measurements or structure refinements. However, the purpose of your study may justify the reported deviations and the more serious of these should normally be commented upon in the discussion or experimental section of a paper or in the "special_details" fields of the CIF. checkCIF was carefully designed to identify outliers and unusual parameters, but every test has its limitations and alerts that are not important in a particular case may appear. Conversely, the absence of alerts does not guarantee there are no aspects of the results needing attention. It is up to the individual to critically assess their own results and, if necessary, seek expert advice.

Publication of your CIF in IUCr journals

A basic structural check has been run on your CIF. These basic checks will be run on all CIFs submitted for publication in IUCr journals (*Acta Crystallographica*, *Journal of Applied Crystallography*, *Journal of Synchrotron Radiation*); however, if you intend to submit to *Acta Crystallographica Section C* or *E* or *IUCrData*, you should make sure that full publication checks are run on the final version of your CIF prior to submission.

Publication of your CIF in other journals

Please refer to the *Notes for Authors* of the relevant journal for any special instructions relating to CIF submission.

PLATON version of 28/11/2022; check.def file version of 28/11/2022

Datablock Pmn21 - ellipsoid plot

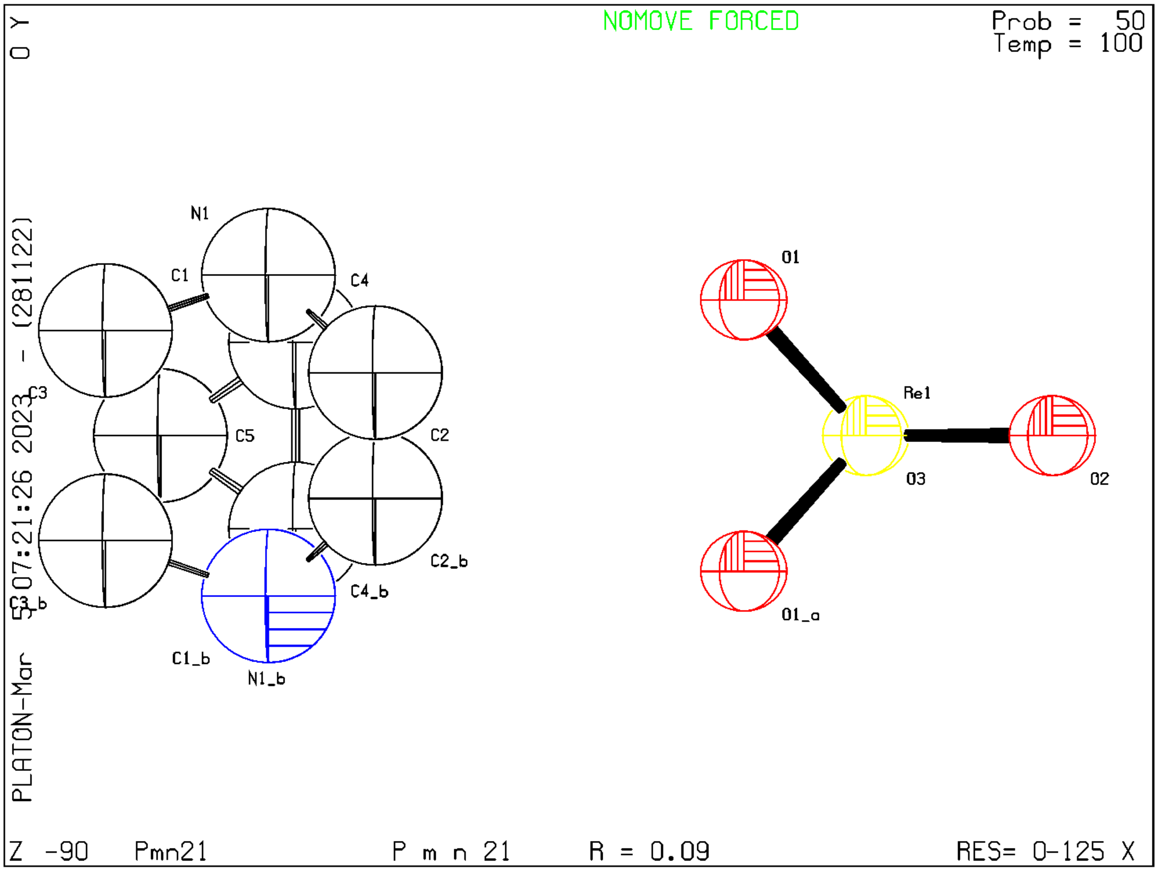

Supplement: Supplementary file 2 — Supporting Information [file ADVS-10-2302426-s002.zip › checkcif for [3.2.1-abco]ReO4 at 100 K.pdf]
