# Supplementary material for: The First Ring Enlargement Induced Large Piezoelectric Response in a Polycrystalline Molecular Ferroelectric
Source: Adv Sci (Weinh). 2023 Jun 16;10(24):2302426. doi: 10.1002/advs.202302426 (PMC10460893; doi:10.1002/advs.202302426)

checkCIF/PLATON report

You have not supplied any structure factors. As a result the full set of tests cannot be run.

THIS REPORT IS FOR GUIDANCE ONLY. IF USED AS PART OF A REVIEW PROCEDURE FOR PUBLICATION, IT SHOULD NOT REPLACE THE EXPERTISE OF AN EXPERIENCED CRYSTALLOGRAPHIC REFEREE.

No syntax errors found. CIF dictionary Interpreting this report

Datablock: PM-3M

```
Bond precision:   = 0.0000 A              Wavelength=1.54178

Cell:               a=6.4362(3)      b=6.4362(3)      c=6.4362(3)
                    alpha=90         beta=90          gamma=90
Temperature:        373 K

                Calculated                  Reported
Volume          266.62(4)                   266.62(4)
Space group     P m -3 m                    P m -3 m
Hall group      -P 4 2 3                    -P 4 2 3
Moiety formula  O4 Re, 0.667(C12)           C8 H0 N0 O4 Re
Sum formula     C8 O4 Re                    C8 H0 N0 O4 Re
Mr              346.29                      346.28
Dx,g cm-3       2.157                       2.157
Z               1                           1
Mu (mm-1)       22.180                      22.180
F000            155.0                       155.0
F000'           150.20
h,k,lmax        8,8,8                       5,7,7
Nref            82                          78
Tmin,Tmax       0.085,0.109                 0.085,0.109
Tmin'           0.024

Correction method= # Reported T Limits: Tmin=0.085 Tmax=0.109
AbsCorr = MULTI-SCAN

Data completeness= 0.951           Theta(max)= 74.939

                                                 wR2(reflections)=
R(reflections)= 0.0409( 61)                      0.1211( 78)

S = 1.063                  Npar= 10
```

The following ALERTS were generated. Each ALERT has the format
test-name_ALERT_alert-type_alert-level.
Click on the hyperlinks for more details of the test.

Alert level C

```
PLAT042_ALERT_1_C Calc. and Reported MoietyFormula Strings  Differ     Please Check
PLAT088_ALERT_3_C Poor Data / Parameter Ratio ...................       8.20 Note
PLAT094_ALERT_2_C Ratio of Maximum / Minimum Residual Density ....      2.88 Report
PLAT260_ALERT_2_C Large Average Ueq of Residue Including       Re1      0.163 Check
PLAT260_ALERT_2_C Large Average Ueq of Residue Including        C1      0.290 Check
```

Alert level G

```
CELLZ01_ALERT_1_G Difference between formula and atom_site contents detected.
CELLZ01_ALERT_1_G ALERT: Large difference may be due to a
             symmetry error - see SYMMG tests
           From the CIF: _cell_formula_units_Z    1
           From the CIF: _chemical_formula_sum  C8 H0 N0 O4 Re
           TEST: Compare cell contents of formula and atom_site data

           atom    Z*formula  cif sites diff
           C          8.00      8.00    0.00
           H          1.00      0.00    1.00
           N          1.00      0.00    1.00
           O          4.00      4.00   -0.00
           Re         1.00      1.00    0.00
PLAT002_ALERT_2_G Number of Distance or Angle Restraints on AtSite          2 Note
PLAT040_ALERT_1_G No H-atoms in this Carbon Containing Compound ..     Please Check
PLAT066_ALERT_1_G Predicted and Reported Tmin&Tmax Range Identical          ? Check
PLAT171_ALERT_4_G The CIF-Embedded .res File Contains EADP Records          1 Report
PLAT172_ALERT_4_G The CIF-Embedded .res File Contains DFIX Records          1 Report
PLAT300_ALERT_4_G Atom Site Occupancy of O1         Constrained at     0.1667 Check
PLAT300_ALERT_4_G Atom Site Occupancy of C1         Constrained at     0.3333 Check
PLAT301_ALERT_3_G Main Residue  Disorder ..............(Resd  1  )        89% Note
PLAT301_ALERT_3_G Main Residue  Disorder ..............(Resd  2  )       100% Note
PLAT304_ALERT_4_G Non-Integer Number of Atoms in ..... (Resd  1  )       4.90 Check
PLAT304_ALERT_4_G Non-Integer Number of Atoms in ..... (Resd  2  )       2.50 Check
PLAT432_ALERT_2_G Short Inter X...Y Contact  C1       ..C1       .       1.90 Ang.
                                                     z,y,-x  =     21_555 Check
PLAT432_ALERT_2_G Short Inter X...Y Contact  C1       ..C1       .       1.90 Ang.
                                                     y,-x,z  =     15_555 Check
PLAT432_ALERT_2_G Short Inter X...Y Contact  C1       ..C1       .       1.90 Ang.
                                                     -z,y,x  =     23_555 Check
PLAT432_ALERT_2_G Short Inter X...Y Contact  C1       ..C1       .       1.90 Ang.
                                                     -y,x,z  =     16_555 Check
PLAT432_ALERT_2_G Short Inter X...Y Contact  C1       ..C1       .       2.11 Ang.
                                                    z,-x,-y  =      6_555 Check
PLAT432_ALERT_2_G Short Inter X...Y Contact  C1       ..C1       .       2.11 Ang.
                                                    y,-z,-x  =     11_555 Check
PLAT432_ALERT_2_G Short Inter X...Y Contact  C1       ..C1       .       2.11 Ang.
                                                    -z,x,-y  =      8_555 Check
PLAT432_ALERT_2_G Short Inter X...Y Contact  C1       ..C1       .       2.11 Ang.
                                                    -y,-z,x  =     12_555 Check
PLAT432_ALERT_2_G Short Inter X...Y Contact  C1       ..C1       .       2.43 Ang.
                                                    -z,-x,y  =      7_555 Check
```

```
PLAT432_ALERT_2_G Short Inter X...Y Contact  C1       ..C1       .       2.43 Ang.
                                                   -y,z,-x  =     10_555 Check
PLAT432_ALERT_2_G Short Inter X...Y Contact  C1       ..C1       .       2.54 Ang.
                                                    -x,z,y  =     18_555 Check
PLAT432_ALERT_2_G Short Inter X...Y Contact  C1       ..C1       .       2.59 Ang.
                                                  -y,-x,-z  =     14_555 Check
PLAT432_ALERT_2_G Short Inter X...Y Contact  C1       ..C1       .       2.59 Ang.
                                                  -z,-y,-x  =     24_555 Check
PLAT432_ALERT_2_G Short Inter X...Y Contact  C1       ..C1       .       2.69 Ang.
                                                   -x,y,-z  =      3_555 Check
PLAT432_ALERT_2_G Short Inter X...Y Contact  C1       ..C1       .       2.69 Ang.
                                                   -x,-y,z  =      2_555 Check
PLAT432_ALERT_2_G Short Inter X...Y Contact  C1       ..C1       .       2.84 Ang.
                                                  -x,-z,-y  =     19_555 Check
PLAT773_ALERT_2_G Check long C-C Bond in CIF: C1       --C1              1.90 Ang.
PLAT773_ALERT_2_G Check long C-C Bond in CIF: C1       --C1              1.90 Ang.
PLAT773_ALERT_2_G Check long C-C Bond in CIF: C1       --C1              1.90 Ang.
PLAT773_ALERT_2_G Check long C-C Bond in CIF: C1       --C1              1.90 Ang.
PLAT811_ALERT_5_G No ADDSYM Analysis: Too Many Excluded Atoms ....          ! Info
PLAT860_ALERT_3_G Number of Least-Squares Restraints ............           1 Note
PLAT883_ALERT_1_G No Info/Value for _atom_sites_solution_primary .     Please Do !
PLAT941_ALERT_3_G Average HKL Measurement Multiplicity ..........         3.3 Low
PLAT950_ALERT_5_G Calculated (ThMax) and CIF-Reported Hmax Differ           3 Units
PLAT965_ALERT_2_G The SHELXL WEIGHT Optimisation has not Converged     Please Check
```

```
   0 ALERT level A = Most likely a serious problem - resolve or explain
   0 ALERT level B = A potentially serious problem, consider carefully
   5 ALERT level C = Check. Ensure it is not caused by an omission or oversight
  39 ALERT level G = General information/check it is not something unexpected

   6 ALERT type 1 CIF construction/syntax error, inconsistent or missing data
  25 ALERT type 2 Indicator that the structure model may be wrong or deficient
   5 ALERT type 3 Indicator that the structure quality may be low
   6 ALERT type 4 Improvement, methodology, query or suggestion
   2 ALERT type 5 Informative message, check
```

Validation response form

Please find below a validation response form (VRF) that can be filled in and pasted into your CIF.

```
# start Validation Reply Form
_vrf_PLAT042_PM-3M
;
PROBLEM: Calc. and Reported MoietyFormula Strings  Differ     Please Check
RESPONSE: ...
;
_vrf_PLAT088_PM-3M
;
PROBLEM: Poor Data / Parameter Ratio ..................       8.20 Note
RESPONSE: ...
;
_vrf_PLAT094_PM-3M
;
PROBLEM: Ratio of Maximum / Minimum Residual Density ....       2.88 Report
RESPONSE: ...
```

```
;
_vrf_PLAT260_PM-3M
;
PROBLEM: Large Average Ueq of Residue Including       Re1      0.163 Check
RESPONSE: ...
;
# end Validation Reply Form
```

It is advisable to attempt to resolve as many as possible of the alerts in all categories. Often the minor alerts point to easily fixed oversights, errors and omissions in your CIF or refinement strategy, so attention to these fine details can be worthwhile. In order to resolve some of the more serious problems it may be necessary to carry out additional measurements or structure refinements. However, the purpose of your study may justify the reported deviations and the more serious of these should normally be commented upon in the discussion or experimental section of a paper or in the "special_details" fields of the CIF. checkCIF was carefully designed to identify outliers and unusual parameters, but every test has its limitations and alerts that are not important in a particular case may appear. Conversely, the absence of alerts does not guarantee there are no aspects of the results needing attention. It is up to the individual to critically assess their own results and, if necessary, seek expert advice.

Publication of your CIF in IUCr journals

A basic structural check has been run on your CIF. These basic checks will be run on all CIFs submitted for publication in IUCr journals (*Acta Crystallographica*, *Journal of Applied Crystallography*, *Journal of Synchrotron Radiation*); however, if you intend to submit to *Acta Crystallographica Section C* or *E* or *IUCrData*, you should make sure that full publication checks are run on the final version of your CIF prior to submission.

Publication of your CIF in other journals

Please refer to the *Notes for Authors* of the relevant journal for any special instructions relating to CIF submission.

PLATON version of 28/11/2022; check.def file version of 28/11/2022

Datablock PM-3M - ellipsoid plot

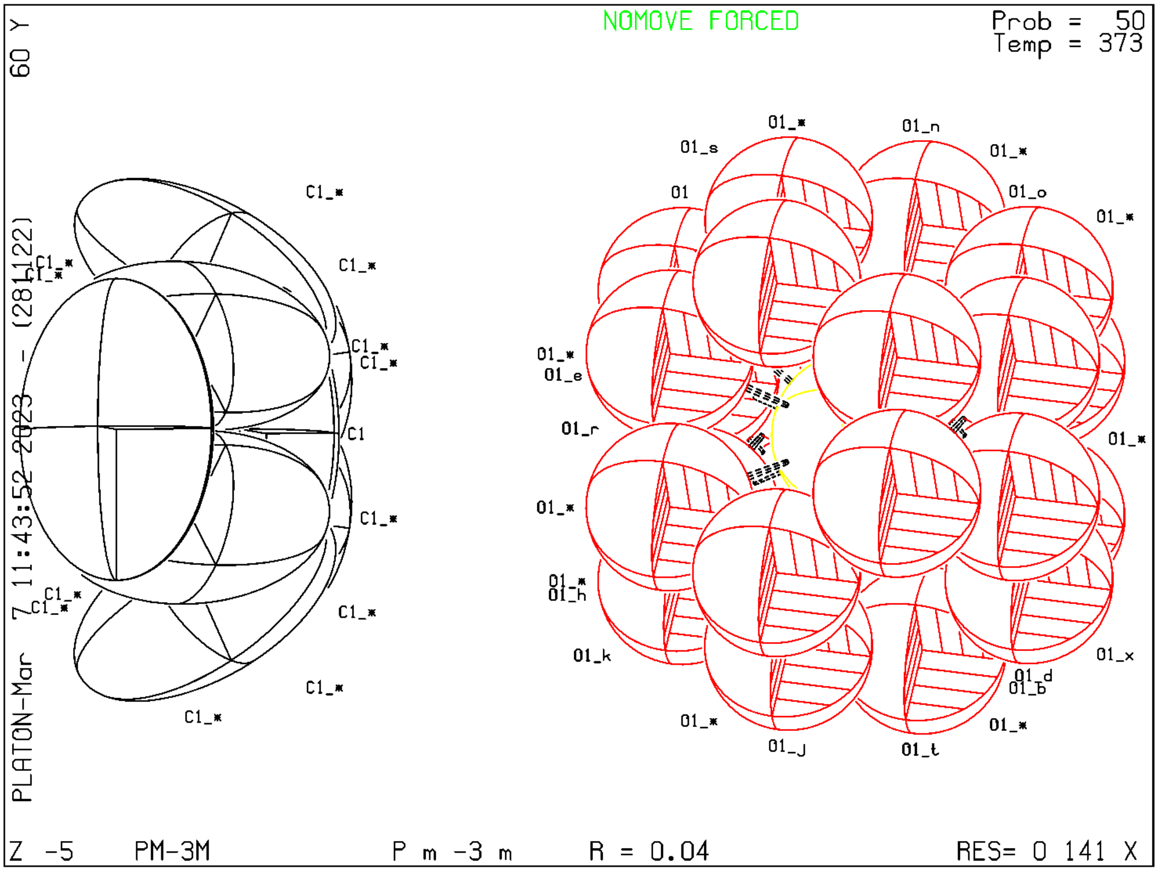

Supplement: Supplementary file 2 — Supporting Information [file ADVS-10-2302426-s002.zip › checkcif for [3.2.1-abco]ReO4 at 373 K.pdf]
