# Supplementary material for: The First Ring Enlargement Induced Large Piezoelectric Response in a Polycrystalline Molecular Ferroelectric
Source: Adv Sci (Weinh). 2023 Jun 16;10(24):2302426. doi: 10.1002/advs.202302426 (PMC10460893; doi:10.1002/advs.202302426)

checkCIF/PLATON report

You have not supplied any structure factors. As a result the full set of tests cannot be run.

THIS REPORT IS FOR GUIDANCE ONLY. IF USED AS PART OF A REVIEW PROCEDURE FOR PUBLICATION, IT SHOULD NOT REPLACE THE EXPERTISE OF AN EXPERIENCED CRYSTALLOGRAPHIC REFEREE.

No syntax errors found.   CIF dictionary   Interpreting this report

Datablock: R3M

```
Bond precision:   = 0.0000 A               Wavelength=1.54178

Cell:             a=6.3552(8)       b=6.3552(8)      c=6.3552(8)
                  alpha=89.7300     beta=89.7300     gamma=89.7300
Temperature:      300 K

                Calculated                    Reported
Volume          256.67(7)                     256.67(10)
Space group     R 3 m r                       R 3 m :R
Hall group      P 3* -2                       P 3* -2
Moiety formula  C7 N, O4 Re                   C7 N, O4 Re
Sum formula     C7 N O4 Re                    C7 H0 N O4 Re
Mr              348.29                        348.28
Dx,g cm-3       2.253                         2.253
Z               1                             1
Mu (mm-1)       23.072                        23.072
F000            156.0                         156.0
F000'           151.21
h,k,lmax        7,7,7                         6,7,7
Nref            413[ 209]                     263
Tmin,Tmax       0.125,0.100                   0.100,0.125
Tmin'           0.020

Correction method= # Reported T Limits: Tmin=0.100 Tmax=0.125
AbsCorr = MULTI-SCAN

Data completeness= 1.26/0.64       Theta(max)= 72.955

                                                      wR2(reflections)=
R(reflections)= 0.0778( 251)                          0.2199( 263)

S = 1.088                   Npar= 34
```

The following ALERTS were generated. Each ALERT has the format
test-name_ALERT_alert-type_alert-level.
Click on the hyperlinks for more details of the test.

Alert level B

```
SYMMS02_ALERT_1_B  Cell angle alpha should be 90 for a trigonal/hexagonal
            cell
  Cell        6.3552    6.3552    6.3552
  Angles     89.7300   89.7300   89.7300
```

Author Response: It is more reasonable to use rhombic unit cell for crystal resolution.

```
SYMMS02_ALERT_1_B  Cell angle beta should be 90 for a trigonal/hexagonal cell
  Cell        6.3552    6.3552    6.3552
  Angles     89.7300   89.7300   89.7300
```

Author Response: It is more reasonable to use rhombic unit cell for crystal resolution.

```
SYMMS02_ALERT_1_B  Cell angle gamma should be 120 for a trigonal/hexagonal
            cell
  Cell        6.3552    6.3552    6.3552
  Angles     89.7300   89.7300   89.7300
```

Author Response: It is more reasonable to use rhombic unit cell for crystal resolution.

Alert level C

```
STRVA01_ALERT_4_C          Flack test results are ambiguous.
           From the CIF: _refine_ls_abs_structure_Flack    0.400
           From the CIF: _refine_ls_abs_structure_Flack_su    0.400
PLAT090_ALERT_3_C Poor Data / Parameter Ratio (Zmax > 18) ........       6.15 Note
PLAT260_ALERT_2_C Large Average Ueq of Residue Including        C1      0.239 Check
PLAT260_ALERT_2_C Large Average Ueq of Residue Including       Re1      0.116 Check
```

Alert level G

```
CELLZ01_ALERT_1_G Difference between formula and atom_site contents detected.
CELLZ01_ALERT_1_G WARNING: H atoms missing from atom site list. Is this intentional?
           From the CIF: _cell_formula_units_Z    1
           From the CIF: _chemical_formula_sum  C7 H0 N O4 Re
           TEST: Compare cell contents of formula and atom_site data

           atom    Z*formula  cif sites diff
           C          7.00      7.00    0.00
           H          1.00      0.00    1.00
           N          1.00      1.00    0.00
           O          4.00      4.00   -0.00
           Re         1.00      1.00    0.00
PLAT002_ALERT_2_G Number of Distance or Angle Restraints on AtSite          7 Note
```

```
PLAT003_ALERT_2_G Number of Uiso or Uij Restrained non-H Atoms ...          7 Report
PLAT032_ALERT_4_G Std. Uncertainty on Flack Parameter Value High .      0.400 Report
PLAT040_ALERT_1_G No H-atoms in this Carbon Containing Compound ..     Please Check
PLAT104_ALERT_1_G The Reported Crystal System is Inconsistent with       R3mR Check
PLAT144_ALERT_4_G s.u. on alpha    Small or Missing .............     0.0000 Degree
PLAT152_ALERT_1_G The Supplied and Calc. Volume s.u. Differ by ...         -3 Units
PLAT171_ALERT_4_G The CIF-Embedded .res File Contains EADP Records          1 Report
PLAT172_ALERT_4_G The CIF-Embedded .res File Contains DFIX Records          8 Report
PLAT177_ALERT_4_G The CIF-Embedded .res File Contains DELU Records          1 Report
PLAT178_ALERT_4_G The CIF-Embedded .res File Contains SIMU Records          1 Report
PLAT186_ALERT_4_G The CIF-Embedded .res File Contains ISOR Records          1 Report
PLAT188_ALERT_3_G A Non-default SIMU Restraint Value has been used     0.0100 Report
PLAT300_ALERT_4_G Atom Site Occupancy of C2         Constrained at        0.5 Check
PLAT300_ALERT_4_G Atom Site Occupancy of N1         Constrained at     0.3333 Check
PLAT300_ALERT_4_G Atom Site Occupancy of O1         Constrained at     0.6667 Check
PLAT300_ALERT_4_G Atom Site Occupancy of O2         Constrained at     0.6667 Check
PLAT301_ALERT_3_G Main Residue  Disorder .............(Resd  1  )        56% Note
PLAT302_ALERT_4_G Anion/Solvent/Minor-Residue Disorder (Resd  2  )        87% Note
PLAT304_ALERT_4_G Non-Integer Number of Atoms in ..... (Resd  2  )       4.17 Check
PLAT773_ALERT_2_G Check long C-C Bond in CIF: C2       --C2              1.90 Ang.
PLAT811_ALERT_5_G No ADDSYM Analysis: Too Many Excluded Atoms ....          ! Info
PLAT860_ALERT_3_G Number of Least-Squares Restraints ............          78 Note
PLAT883_ALERT_1_G No Info/Value for _atom_sites_solution_primary .     Please Do !
PLAT941_ALERT_3_G Average HKL Measurement Multiplicity ...........        2.8 Low
```

---

 0 **ALERT level A** = Most likely a serious problem - resolve or explain
 3 **ALERT level B** = A potentially serious problem, consider carefully
 4 **ALERT level C** = Check. Ensure it is not caused by an omission or oversight
27 **ALERT level G** = General information/check it is not something unexpected

 9 ALERT type 1 CIF construction/syntax error, inconsistent or missing data
 5 ALERT type 2 Indicator that the structure model may be wrong or deficient
 5 ALERT type 3 Indicator that the structure quality may be low
14 ALERT type 4 Improvement, methodology, query or suggestion
 1 ALERT type 5 Informative message, check

It is advisable to attempt to resolve as many as possible of the alerts in all categories. Often the minor alerts point to easily fixed oversights, errors and omissions in your CIF or refinement strategy, so attention to these fine details can be worthwhile. In order to resolve some of the more serious problems it may be necessary to carry out additional measurements or structure refinements. However, the purpose of your study may justify the reported deviations and the more serious of these should normally be commented upon in the discussion or experimental section of a paper or in the "special_details" fields of the CIF. checkCIF was carefully designed to identify outliers and unusual parameters, but every test has its limitations and alerts that are not important in a particular case may appear. Conversely, the absence of alerts does not guarantee there are no aspects of the results needing attention. It is up to the individual to critically assess their own results and, if necessary, seek expert advice.

Publication of your CIF in IUCr journals

A basic structural check has been run on your CIF. These basic checks will be run on all CIFs submitted for publication in IUCr journals (*Acta Crystallographica*, *Journal of Applied Crystallography*, *Journal of Synchrotron Radiation*); however, if you intend to submit to *Acta Crystallographica Section C* or *E* or *IUCrData*, you should make sure that full publication checks are run on the final version of your CIF prior to submission.

Publication of your CIF in other journals

Please refer to the *Notes for Authors* of the relevant journal for any special instructions relating to CIF submission.

PLATON version of 28/11/2022; check.def file version of 28/11/2022

Datablock R3M - ellipsoid plot

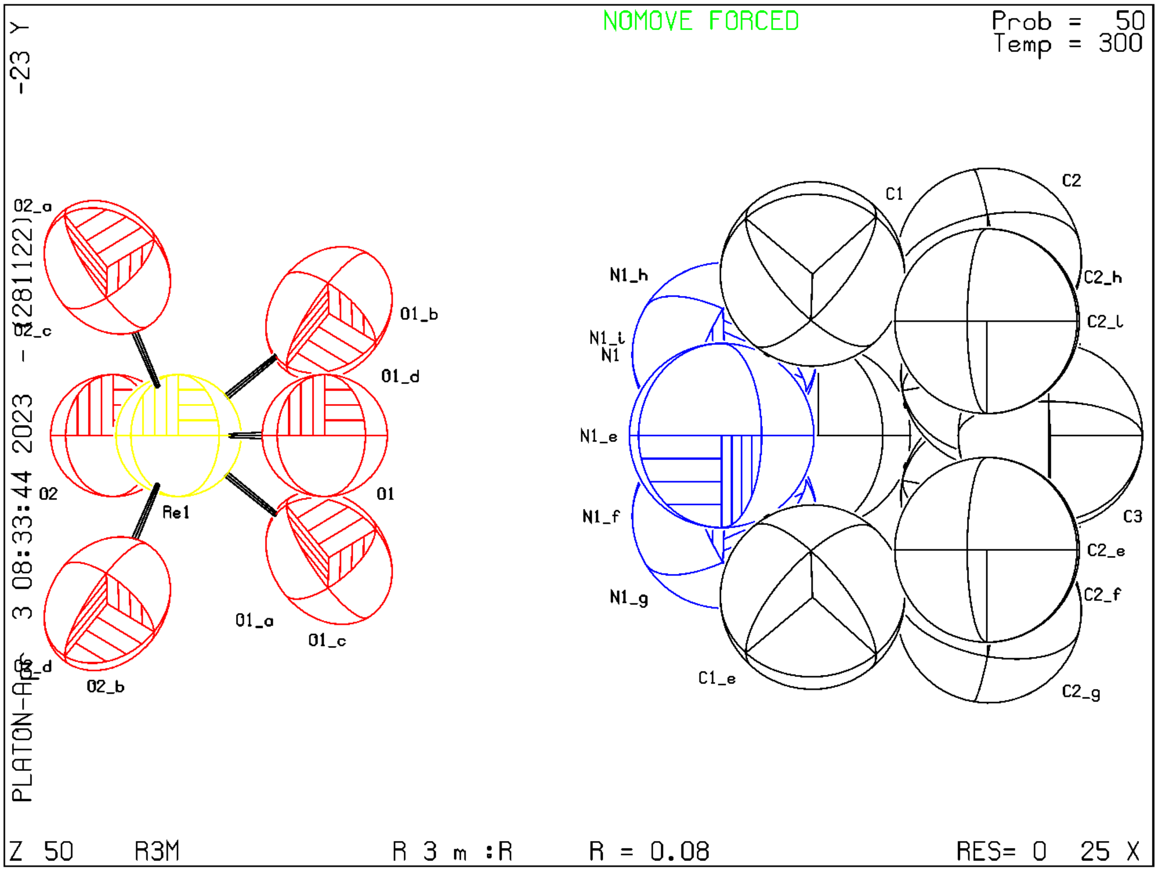

Supplement: Supplementary file 2 — Supporting Information [file checkcif for [3.2.1-abco]ReO4 at 300 K.pdf › checkcif for [3.2.1-abco]ReO4 at 300 K.pdf]
